# Asymmetric purine-pyrimidine distribution in cellular small RNA population of papaya

**DOI:** 10.1186/1471-2164-13-682

**Published:** 2012-12-05

**Authors:** Rishi Aryal, Xiaozeng Yang, Qingyi Yu, Ramanjulu Sunkar, Lei Li, Ray Ming

**Affiliations:** 1 Department of Plant Biology, University of Illinois at Urbana-Champaign, Urbana, IL, 61801, USA; 2 Department of Biology, University of Virginia, Charlottesville, VA, 22904, USA; 3 Texas AgriLife Research Center, Department of Plant Pathology and Microbiology, Texas A&M University, Weslaco, TX, 78596, USA; 4 Department of Biochemistry and Molecular Biology, Oklahoma State University, Stillwater, OK, 74078, USA

**Keywords:** miRNA, siRNA, Papaya Ringspot Virus (PRSV), Small RNA strand selection, Transgene silencing

## Abstract

**Background:**

The small RNAs (sRNA) are a regulatory class of RNA mainly represented by the 21 and 24-nucleotide size classes. The cellular sRNAs are processed by RNase III family enzyme dicer (Dicer like in plant) from a self-complementary hairpin loop or other type of RNA duplexes. The papaya genome has been sequenced, but its microRNAs and other regulatory RNAs are yet to be analyzed.

**Results:**

We analyzed the genomic features of the papaya sRNA population from three sRNA deep sequencing libraries made from leaves, flowers, and leaves infected with Papaya Ringspot Virus (PRSV). We also used the deep sequencing data to annotate the micro RNA (miRNA) in papaya. We identified 60 miRNAs, 24 of which were conserved in other species, and 36 of which were novel miRNAs specific to papaya. In contrast to the Chargaff’s purine-pyrimidine equilibrium, cellular sRNA was significantly biased towards a purine rich population. Of the two purine bases, higher frequency of adenine was present in 23nt or longer sRNAs, while 22nt or shorter sRNAs were over represented by guanine bases. However, this bias was not observed in the annotated miRNAs in plants. The 21nt species were expressed from fewer loci but expressed at higher levels relative to the 24nt species. The highly expressed 21nt species were clustered in a few isolated locations of the genome. The PRSV infected leaves showed higher accumulation of 21 and 22nt sRNA compared to uninfected leaves. We observed higher accumulation of miRNA* of seven annotated miRNAs in virus-infected tissue, indicating the potential function of miRNA* under stressed conditions.

**Conclusions:**

We have identified 60 miRNAs in papaya. Our study revealed the asymmetric purine-pyrimidine distribution in cellular sRNA population. The 21nt species of sRNAs have higher expression levels than 24nt sRNA. The miRNA* of some miRNAs shows higher accumulation in PRSV infected tissues, suggesting that these strands are not totally functionally redundant. The findings open a new avenue for further investigation of the sRNA silencing pathway in plants.

## Background

Small RNAs (sRNAs) are a regulatory class of RNAs present in broad range of eukaryotic organisms and some viruses. Micro RNA (miRNA), small interfering RNA (siRNA), and natural antisense siRNA are the major regulatory RNAs in plants. They are processed by RNase III domain containing protein of dicer family (Dicer like in plants). Another major class of regulatory RNA, Piwi-interacting RNA (piRNA), targets transposable elements in animal genomes [1]. The dicer processed RNA duplex is incorporated into RNA induced silencing complex (RISC) containing the RNase H class ribonuclease, Argonaute, which carries out the regulatory functions in a sequence specific manner. After incorporation to the RISC complex, one of the two strands is selected as an effector molecule, called the guide strand, by a mechanism not yet known. The complementary strand, called the passenger strand, has no known function and is degraded by cellular machinery. The guide strands are involved in posttranscriptional gene silencing in a spatiotemporal manner. Some sRNAs are also implicated in transcriptional gene silencing by chromatin modification [2-8].

Cellular sRNAs are mainly expressed from repetitive sequences, intergenic regions, and introns of genes [9-12]. Some effort has been made to characterize *cis* regulatory motifs on the genomic loci expressing small regulatory RNAs [13,14]. Genomic characterization of the loci and their regulatory motifs will provide useful information to understand the biology of these regulatory RNAs. High throughput deep sequencing data has been used to analyze the vast amount of sRNA populations in plants [15-20]. Most of these studies focus on characterizing miRNA and finding their targets. The micro RNAs are transcribed from more canonical genetic structures, having promoters identified by RNA polymerase II [21-25]. In several cases, the altered or mis-expression of miRNA shows a distinct phenotype and is thus easier to discover and characterize. On the other hand, a bigger portion of the cellular sRNA population is made of non-microRNA class. Genomic and transcriptomic features of a large number of these potentially regulatory sRNAs are yet to be fully characterized.

Micro RNA is a class of small regulatory RNA that functions as a negative regulator of target mRNA. They are processed from a single primary transcript that folds back, forming a stable stem-loop structure. Most miRNAs play a key role in controlling various developmental events [4,26-30], or are associated with response to different biotic and abiotic stimuli [31-37]. The miRNAs are relatively conserved across diverse plant species and have definite evolutionary history among plant and animal kingdoms [38-42]. Annotation and functional analysis of miRNA from more organisms are needed for detailed understanding of their evolutionary prospective and functional importance in the cell.

Papaya is emerging as a model species to study sex chromosome evolution in plants and also for tropical fruit tree genomics [43]. A 271Mb draft genome of papaya covering 73% of the total genome (372Mb) and 92% of the euchromatic region is available [44]. The genome contains 52% of the repetitive regions and the total GC content of the genome is 35.3%. The genome has not gone through whole genome duplication after the ancient triplication event. Papaya contains the minimal set of protein coding genes among all sequenced angiosperm species. Expression of a miRNA and some putative regulatory RNAs in papaya has been reported before [45]. The complete profiling of small regulatory RNAs is still lacking. Furthermore, the sequenced cultivar SunUp is the transgenic line containing coat protein of Papaya Ringspot Virus (PRSV) to develop resistance against the virus [46,47]. This provides an opportunity for study of virus resistant mechanisms in transgenic plants. We present the detailed analysis of the cellular sRNA population from papaya and discuss the significance of purine-pyrimidine bias observed in the population. We compared the total sRNA population in three papaya tissues and in transgenic and non-transgenic lines. We further used the sRNA sequences to annotate the miRNA genes in papaya and analyzed the expression pattern in different tissues, including PSRV infected leaves.

## Results

Three sRNA libraries prepared from SunUp leaves, AU9 female flowers, and AU9 male leaves infected with PRSV were sequenced using the Illumina GAII system. After adapter trimming, 18-33nt reads were extracted and 18-26nt sequences were taken as small regulatory RNA and used for further analysis. A total of 4,657,833 reads were obtained from female flowers, 4,664,779 read were obtained from leaves, and 4,505,266 reads were obtained from PRSV infected leaves (Additional file 1: Table S1). The sequences constitute 2,200,544 (47.24%), 2,033,600 (43.59%), and 1,288,216 (28.59%) unique reads in the flowers, leaves, and PRSV infected leaves, respectively.

**Figure 1:**
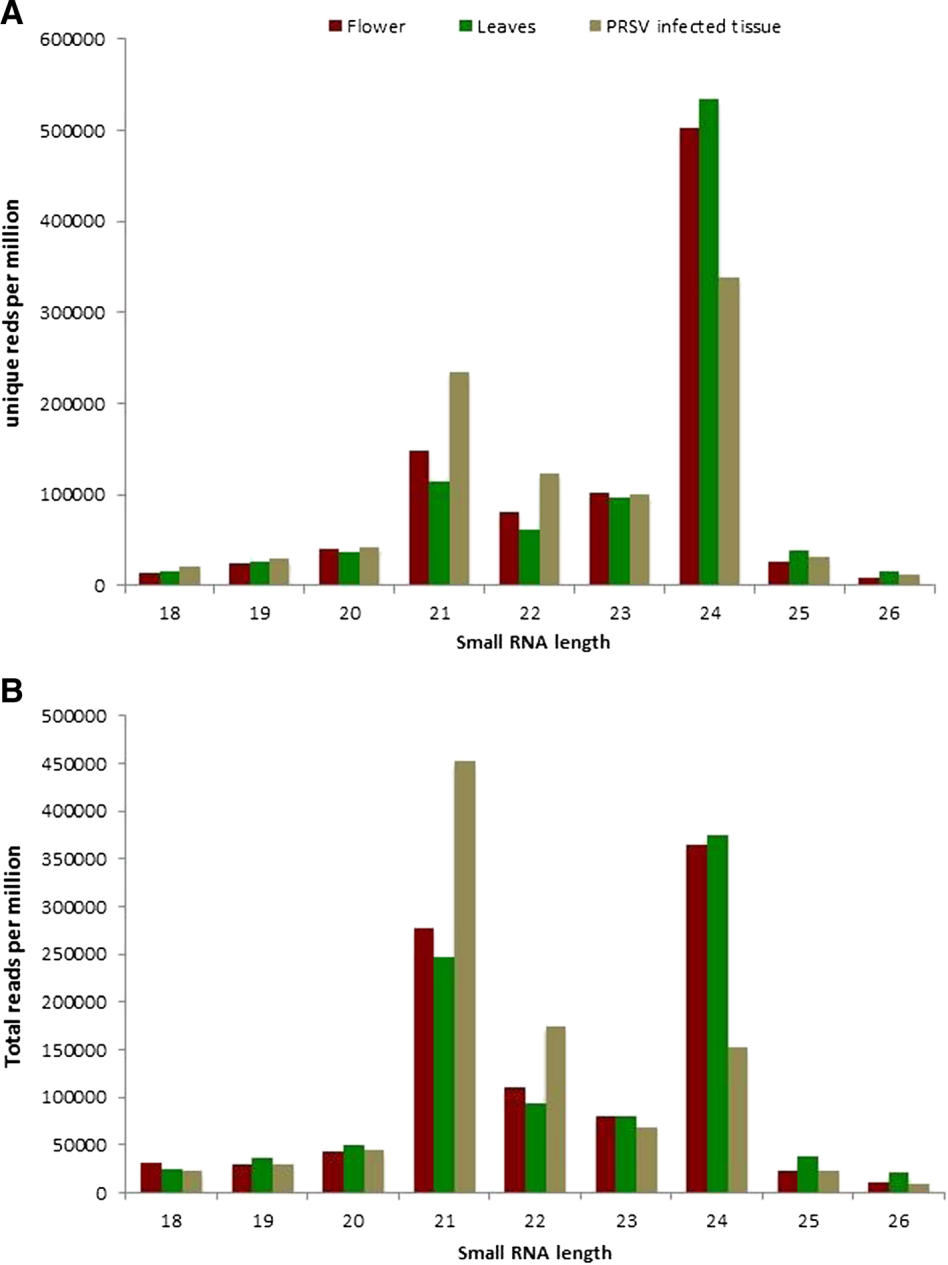
**PRSV infection causes higher expression of 21 and 22nt sRNAs in papaya. A**. Size distribution of unique populations. **B**. Size distribution of total redundant reads.

We compared the different size classes of sRNAs in three libraries. As expected, the 21 and 24nt species were the major constituents in all samples (Figure 1A). Comparison of the unique reads to the total redundant reads showed that the total 21 and 22nt sequences are more expressed relative to 24nt species. The ratio of 24nt to 21nt species unique reads were 3.3, 4.6, and 1.4 for flowers, leaves, and PRSV infected leaves, whereas this ratio was 1.3, 1.5 and 0.33 in total redundant reads. The 21 and 22nt unique sequences in PRSV infected leaves were expressed higher compared to that of the uninfected leaves, whereas the 24nt species showed the opposite trend (Figure 1B). Comparing the reads specific to the three libraries, the number of 21 and 22nt reads specific to PRSV infected leaves was significantly higher than the uninfected leaves. On the other hand, the 24nt reads specific to PRSV infected leaves were much lower compared to uninfected tissues (Additional file 1: Figure S1).

### Annotation of papaya miRNAs

A total of 60 miRNAs from 53 families along with their miRNA* sequences were identified from the three sets of sRNA deep sequencing reads (Table 1, Additional file 1: Table S2). The miRNAs were identified based on stem-loop structure using algorithm miRDeep [48] optimized for plant miRNA prediction [49] with the optimal precursor length of 250nt. The miRDeep algorithm calls miRNA from the aligned reads only when it finds both guide (miRNA) and star (miRNA*) strands in the library and they can form a stable hairpin structure, making it the most robust program to identify miRNA from the deep sequencing reads. Of the 60 miRNAs annotated in papaya, 24 show strong homology to previously annotated miRNAs from other plant species, while 36 appear to be specific to papaya. Out of the 60 annotated miRNAs, 18 miRNAs were only detected in flowers, 12 only in leaves, and five were only in PRSV infected leaves. The expression of the predicted miRNAs were tested by stem-loop qRT-PCR assay [50] (Additional file 1: Figure S2). A total of 62 miRNAs, including nine miRNA*, were tested for their expression. Of these, 60 were detected in at least one library, while two were not detected in all three libraries.

**Table 1:** Expression analysis of Papaya Micro RNAs

	**MiR ID**	**qPCR fold change**			**Deep sequencing reads per million**		
		**Flower**	**Leaves**	**PRSV infected leaves**	**Flower**	**Leaves**	**PRSV infected leaves**
1	cpa-MIR156/57a	1.00	1.46	2.70	132.68	2426.48	1093.61
2	cpa-MIR156/57b	1.00	0.09	0.03	132.47	815.04	540.48
3	cpa-MIR159a	1.00	0.80	0.05	441.84	805.61	0.00
4	cpa-MIR159b^†^	1.00	0.06	0.03	0.00	0.00	0.67
5	cpa-MIR159/319^†^				1.07	0.00	0.00
6	cpa-MIR160	1.00	0.18	0.46	19.32	16.08	11.32
	cpa-MIR160*	1.00	0.32	27.34	0.00	0.00	191.11
7	cpa-MIR164	1.00	0.10	0.25	229.94	114.05	77.69
8	cpa-MIR165/166a^††^	1.00	1.20	0.70	16800.30	29790.48	21150.36
9	cpa-MIR165/166b^††^				4096.54	8447.99	6703.93
11	cpa-MIR165/166c^††^				0.00	161.8	0.00
10	cpa-MIR166b*	1.00	0.47	29.51	0.00	0.00	456.35
	cpa-MIR166c*				0.00	83.60	0.00
12	cpa-MIR167a^†††^	1.00	1.17	2.30	171.54	155.63	35.74
13	cpa-MIR167b^†††^				60.11	650.83	176.68
14	cpa-MIR167c^†††^				17.60	4725.63	1581.26
	cpa-MIR167*	1.00	1.13	0.46	0.00	168.50	0.00
15	cpa-MIR169	1.00	0.19	0.26	0.00	0.00	0.44
16	cpa-MIR170/71	1.00	0.41	0.69	22.97	94.32	35.96
17	cpa-MIR172	1.00	0.07	0.13	5.58	0.00	0.00
18	cpa-MIR390	1.00	0.48	1.14	428.53	0.00	0.00
	cpa-MIR390*	1.00	0.12	0.31	0.00	0.00	8.88
19	cpa-MIR393	1.00	0.57	0.43	1.50	0.00	0.00
	cpa-MIR393*	1.00	0.42	6.79	0.00	0.00	11.10
20	cpa-MIR394	1.00	0.70	0.57	167.67	202.80	116.31
21	cpa-MIR395	1.00	1.36	0.54	0.00	1.50	7.10
22	cpa-MIR396	1.00	2.63	0.15	0.00	55.74	0.00
	cpa-MIR396*	1.00	0.72	5.25	79.01	0.00	1021.03
23	cpa-MIR408	1.00	0.11	0.15	9.23	0.00	0.00
	cpa-MIR408*	1.00	0.16	12.41	0.00	4.07	45.72
24	cpa-MIR535	1.00	0.51	0.23	292.63	686.85	348.48
25	cpa-MIR-novel_01	1.00	0.60	0.71	13.31	30.87	9.10
26	cpa-MIR-novel_02	1.00	0.15	1.43	225.86	69.46	3595.35
27	cpa-MIR-novel_03	1.00	0.22	30.76	8.37	8.15	19.31
28	cpa-MIR-novel_04	1.00	0.19	0.36	3.01	6.43	3.33
29	cpa-MIR-novel_05	1.00	0.20	0.82	110.14	134.84	21.75
30	cpa-MIR-novel_06	1.00	0.11	0.52	54.10	40.52	0.00
	cpa-MIR-novel_06*	1.00	0.19	284.64	0.00	0.00	108.10
31	cpa-MIR-novel_07	1.00	0.15	4.12	3.44	0.00	1.55
32	cpa-MIR-novel_08	1.00	0.22	0.16	0.00	1.29	0.00
33	cpa-MIR-novel_09a	1.00	0.30	0.29	0.00	0.64	0.00
34	cpa-MIR-novel_09b	1.00	0.45	0.44	0.00	13.51	29.30
35	cpa-MIR-novel_10	1.00	2.15	1.67	71.71	0.00	0.00
36	cpa-MIR-novel_11	1.00	0.73	0.44	57.75	0.00	0.00
37	cpa-MIR-novel_12	1.00	0.02	0.14	37.36	0.00	0.00
38	cpa-MIR-novel_13	1.00	0.53	0.63	6.44	0.00	0.00
39	cpa-MIR-novel_14	1.00	0.28	0.00	5.37	0.00	0.00
40	cpa-MIR-novel_15	1.00	0.29	0.28	4.29	0.00	0.00
41	cpa-MIR-novel_16	1.00	0.28	0.56	3.01	0.00	0.00
42	cpa-MIR-novel_17	1.00	0.00	0.51	2.58	0.00	0.00
43	cpa-MIR-novel_18	1.00	0.87	2.52	2.58	0.00	0.00
44	cpa-MIR-novel_19	1.00	0.24	0.65	2.58	0.00	0.00
45	cpa-MIR-novel_20	Not detected by qPCR			4.07	0.00	0.00
46	cpa-MIR-novel_21	1.00	0.29	0.24	2.36	0.00	0.00
47	cpa-MIR-novel_22	1.00	1.69	0.29	2.15	0.00	0.00
48	cpa-MIR-novel_23	1.00	0.28	0.19	2.15	0.00	0.00
49	cpa-MIR-novel_24	1.00	0.13	0.00	0.00	12.22	0.00
50	cpa-MIR-novel_25	1.00	0.14	0.00	0.00	9.00	0.00
51	cpa-MIR-novel_26	1.00	0.37	0.48	0.00	5.57	0.00
52	cpa-MIR-novel_27	1.00	1.00	0.10	0.00	4.07	0.00
53	cpa-MIR-novel_28	Not detected by qPCR			0.00	2.36	0.00
54	cpa-MIR-novel_29	1.00	0.04	0.11	0.00	3.22	0.00
55	cpa-MIR-novel_30	1.00	0.42	0.22	0.00	3.00	0.00
56	cpa-MIR-novel_31	1.00	0.21	0.88	0.00	2.79	0.00
57	cpa-MIR-novel_32	1.00	0.17	0.40	0.00	2.14	0.00
58	cpa-MIR-novel_33	1.00	6.94	5.25	0.00	0.00	6.66
59	cpa-MIR-novel_34	1.00	0.00	2.06	0.00	0.00	6.66
60	cpa-MIR-novel_35	0.00	1.00	3.60^¥^	0.00	0.00	2.44

qPCR fold change is normalized on initial RNA input, the sequencing expression level was normalized on per million reads of respective library.

†,††,††† - The sequences can not be distinguished by stem-loop primers, so tested together for qPCR.

¥ - normalized on leaf expression level since no expression detected on flower.

**Figure 2:**
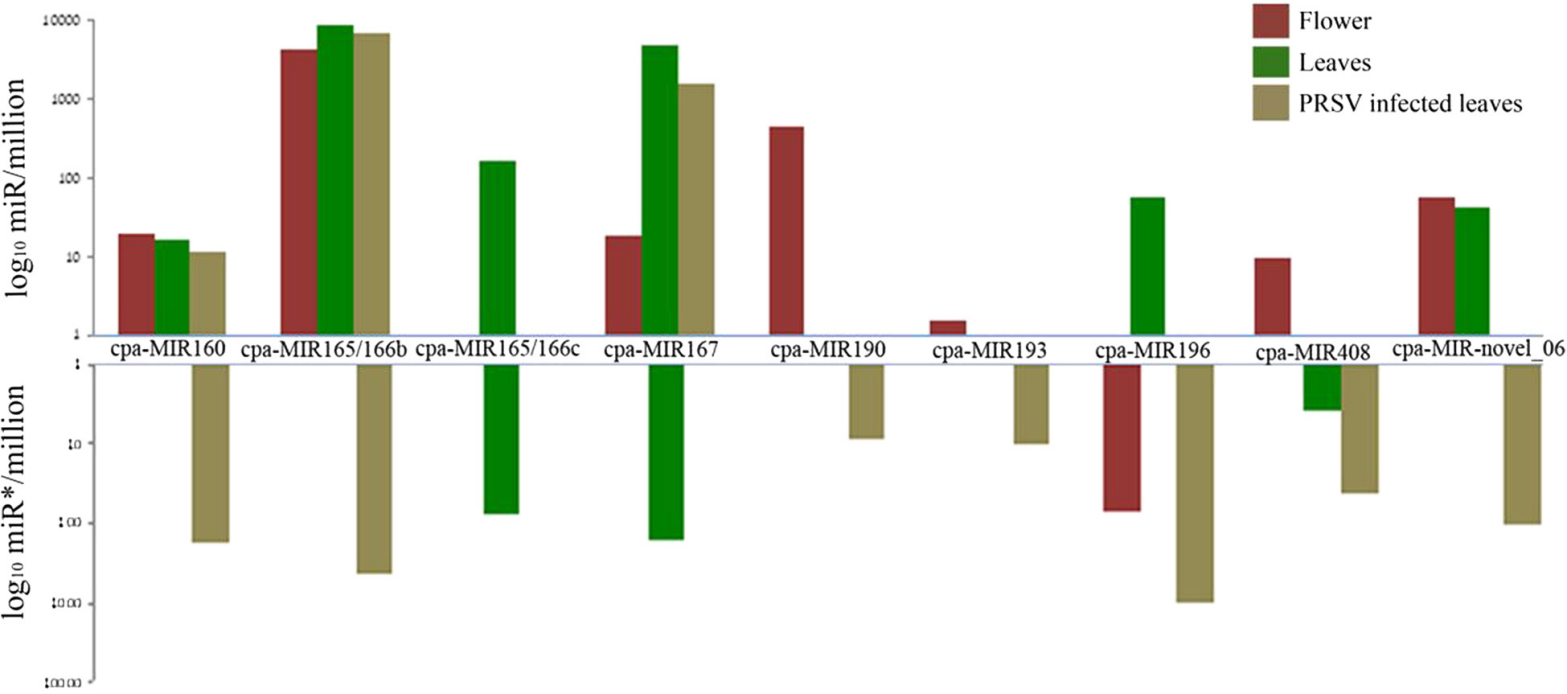
miRA* is accumulated in the virus infected tissue. The y-axis represents the reads per million in respective libraries on a log10 scale. The bar above the base line represents the guide strand and the bar below the base line represents the passenger strand in each library.

Of the 53 miRNA families identified in papaya, the miRNAs* of nine miRNAs were detected at a higher levels than their respective miRNA. The accumulation of the miRNA* varied from nine reads per million (miR390*) to 1021 reads per million (miR396*). The higher accumulation of the miRNA* was mostly observed in PRSV infected leaves (Figure 2, Table 1). Of the nine miRNA families showing higher miRNA* accumulation than respective miRNA, seven showed higher levels in PRSV infected leaves compared to uninfected leaves. The remaining two families showed higher accumulation in leaf tissue (Figure 2).

### Majority of cellular sRNA is represented by only one copy

**Figure 3:**
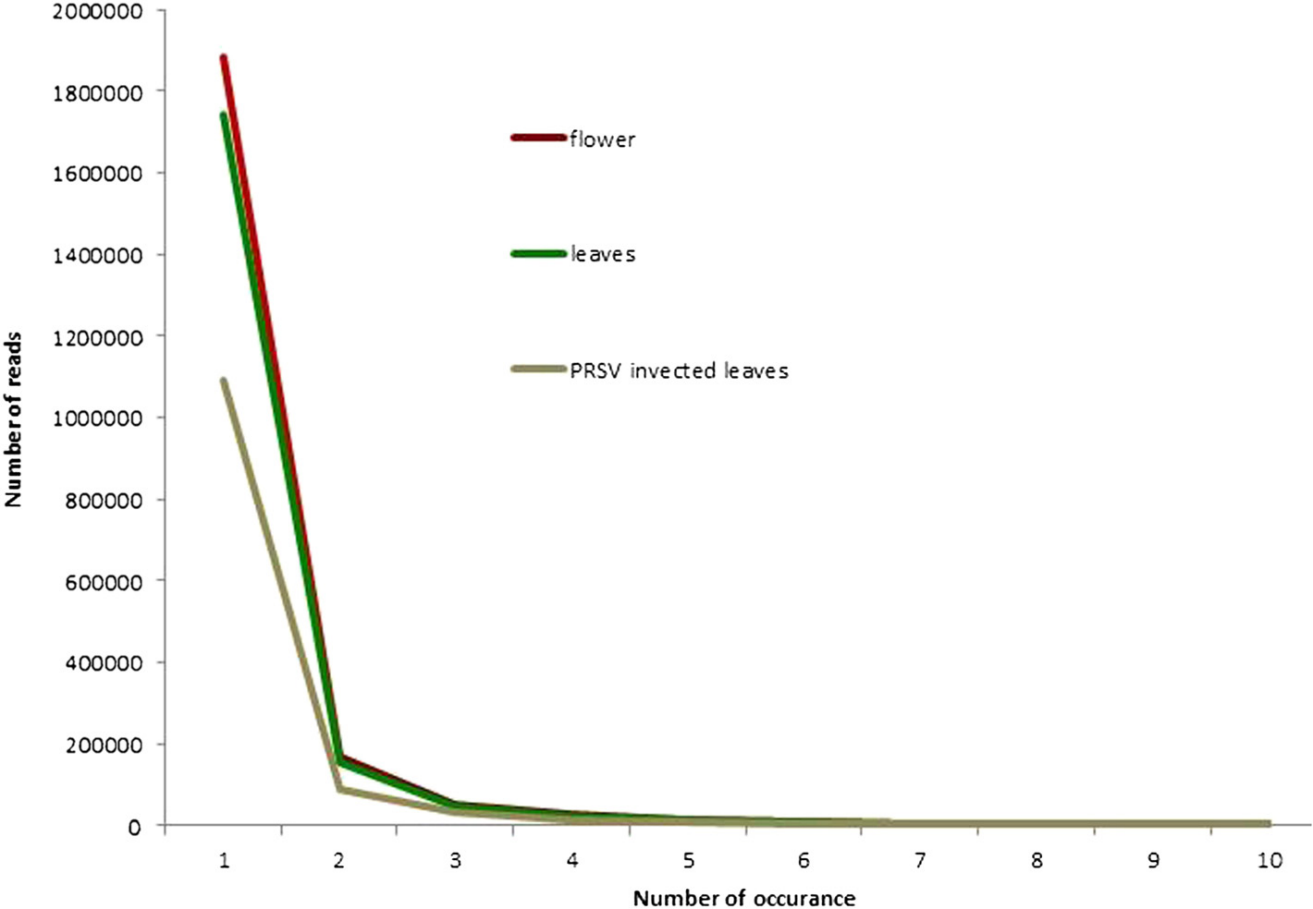
**Majority of the sRNA population in the cell are represented by only one copy. **The y-axis represents the number of unique reads and the x-axis shows the number occurrences in each library. The reads occurring more than 10 times are not shown in the figure as they follow the same trend after 10.

We analyzed the abundance of the unique sRNA reads in each library (Figure 3). A large number of reads were represented by only one copy in the entire library. Single copy reads constituted 85.3%, 85.6%, and 84.7% of unique reads in flowers, leaves, and PRSV infected leaves, while reads with over 10 copies constituted only 1.4%, 1.2%, and 2.3% of the unique reads. Most of the single copy sRNAs differ from one another by a few nucleotides and map to overlapping genomic loci (Additional file 1: Figure S3). We checked the proportion of single copy reads in sRNA datasets from 6 plant species, *Arabidopsis thaliana, Populus trichocarpa, Medicago truncatula, Arachis hypogea, Glycine max, and Phaseolous Vulgaris,* obtained from NCBI’s gene expression omnibus. The single copy reads in these plant species ranged from 73.6% (*A. thaliana*) to 90% (*M. truncatula*) (Additional file 1: Figure S4).

### Mapping sRNA to the papaya genome

The sRNA reads were mapped to the papaya draft genome [44]. The percentage of unique reads showing a perfect match to the genome were 55.0%, 57.8%, and 54.4% from the flower, leaves, and PRSV infected leaves respectively. The papaya draft genome contains 271 Mb constituting 73% of the total genome size (372 Mb). Approximately 45% of the unmapped reads should be coming from the remaining 27% highly repetitive region of the genome not represented in the draft genome [44].

**Figure 4:**
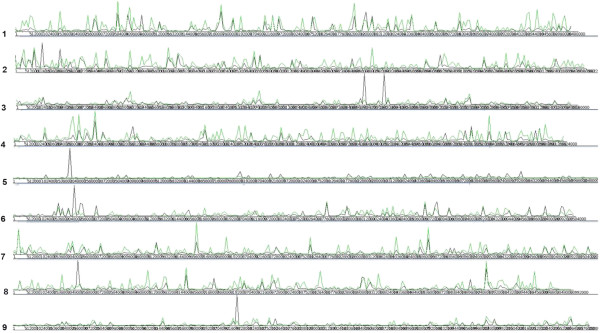
**Mapping 21nt and 24nt reads to the genome shows higher expression of 21nt sequences from some genomic islands while 24nt sequences are evenly distributed throughout the genome. **The horizontal bar represents nine unordered papaya chromosomes, the chromosome number denoted by the figure at the left. The black lines represent 21nt reads and the green lines represent 24nt reads.

Different size class sRNA transcripts shows distinct nucleotide composition (see next section), implying their different genomic location of origin. It has been reported that 24nt sRNAs are more or less evenly expressed throughout the genome, while 21nt sRNAs show higher expression from some discrete genomic regions [15]. To characterize the genomic regions expressing different size class sRNA in papaya, we mapped the 21nt and 24nt reads from three libraries to the nine papaya chromosomes [51] (Figure 4). The 24nt reads were mapped evenly throughout the genome and have higher expression than the 21nt species in most genomic loci, whereas the 21nt reads showed much higher expression than the 24nt species in some isolated regions of the genome (Chromosomes 3, 5, 6, 8, and 9 in Figure 4).

### Cellular sRNA shows accumulation of purine rich strands

**Figure 5:**
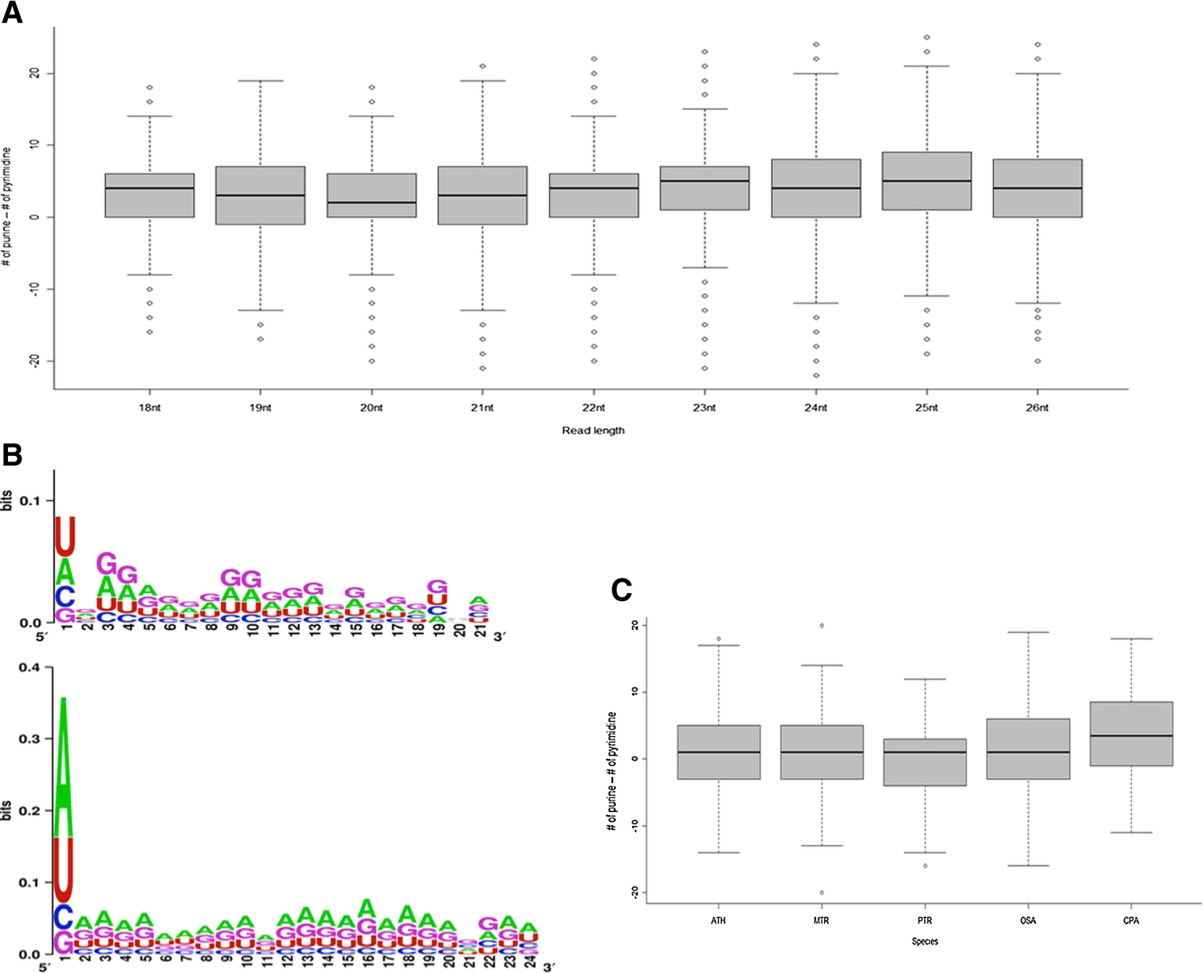
**Endogenous sRNA populations are overrepresented by purine rich sequences but miRNAs do not show this bias. A**. Distribution of purine rich and pyrimidine rich papaya sRNA population. The reads above zero are rich in purine and below zero are rich in pyrimidine. **B**. Weblogo representation of randomly picked 10,000 sRNA reads of 21nt and 24nt. The Y-axis represents the bit score, which can be a maximum of 4 for complete conservation of a nucleotide. The letter on the top is present highest in the sample. **C**. Distribution of purine and pyrimidine in annotated miRNAs from miRBASE in 4 species *(Arabidopsis thaliana, Medicago truncatula, Populous trichocarpa, Oriza sativa, and Carica papaya).*

Since the endogenous sRNAs are processed from a double stranded precursor, an equal ratio of purine-pyrimidine bases in the population is expected based on Chargaff’s rule. Interestingly, the analysis of endogenous sRNAs in all papaya libraries showed significant deviation towards purine rich sequences (Fisher’s exact test; p=10^-4^) (Figures 4B and 5A). Approximately 75% of the unique reads in the dataset were higher in purine residues than pyrimidine residues. To check whether this bias was coming from some specific position, we analyzed the frequency of each nucleotide on every nucleotide position of the sRNA sequences. The two purine bases, adenine and guanine, were the most frequent at each nucleotide position, followed by the pyrimidines, uracil and cytosine. Of the two purine nucleotides, the frequency of guanine was highest in 21nt sequences while the adenine was the most frequent in 24nt species (Figure 5B). While the percentage of cytosine and uracil remains the same in both 21 and 24nt species, percentage of guanine decreased from 27.9 in 21nt species to 25.2 in 24nt species and the adenine component increased from 29.5 in 21nt species to 33.6 in 24nt species. The 5’ nucleotide of the 24nt species was biased towards adenine while uracil was most frequent nucleotide at the 5’ end of 21nt species, as reported previously [15,52]. The 5’ nucleotide conservation on 24nt species was more prominent than on 21nt species (~0.3 bit in 24nt and ~0.1 bit in 21nt in the scale of zero bit for no conservation and 2.0 bit for complete conservation) (Figure 5B).

To examine whether this observed biased purine-pyramidine distribution is specific to papaya sRNAs or a general phenomena, we analyzed the nucleotide composition in sRNA datasets of six more plant species from NCBI’s gene expression omnibus, *A. thaliana, P. trichocarpa, M. truncatula, A. hypogea, G. max, and P. vulgaris*. All six species analyzed showed the overabundance of purine rich sequences in the population compared to pyrimidine rich sequences. The difference between purine rich sequences and pyrimidine rich sequences was observed in each of the 18-25nt sequences of Arabidopsis (Additional file 1: Figure S5A and S5B). Consistent with papaya data, the shift from high frequency guanine in 21nt species to high frequency adenine in 24nt species was observed in all plants species analyzed.

We analyzed the purine-pyrimidine composition in the annotated miRNA sequences in 5 plant species. A total of 329 miRNAs from Arabidopsis, 675 from *Medicago truncatula*, 238 from *Populus trichocarpa,* and 662 from *Oryza sativa* in MirBase [53], and 60 miRNAs from papaya (from this study) were analyzed for purine-pyrimidine composition. Although the purine rich sequences were consistently higher in all species, the difference was not as prominent as observed in total sRNA population (Figure 5C). No definable pattern of nucleotide frequency was observed along the miRNA sequences as was observed in total sRNA population (Additional file 1: Figure S6). This may be due to the evolutionary history of the miRNAs (see discussion).

### Viral small RNA and transgene silencing

**Figure 6:**
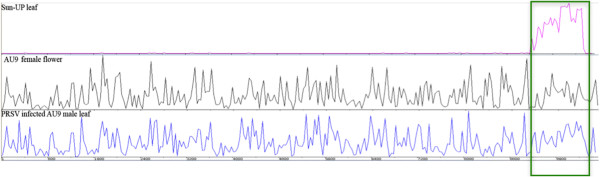
**The transgenic papaya produces sRNAs corresponding to the transgene region of the viral genome while in non-transgenic plant the sRNAs come from throughout the viral genome. **Map showing alignment of small RNA from three papaya libraries on Papaya Ringspot Virus (PRSV) genome. The x-axis shows the 10326bp PRSV genome. SunUp papaya is transgenic cultivar transformed with coat protein gene of the PRSV, AU9 is non-transtgenic cutivar. The green box shows the coat protein region of the PRSV genome.

SunUp cultivar of papaya was transformed with coat protein gene from the PRSV to develop the virus resistant lines [46,47]. Integration of PRSV coat protein gene of the virus in the papaya genome has been confirmed [44]. The AU9 cultivar on the other hand is non-transgenic and susceptible to PRSV infection. The sRNA reads from three libraries were aligned to the PRSV genome (NCBI Reference Sequence: NC_001785.1). A total of 1,915 reads from SunUp leaves and 19,531 from the PRSV infected AU9 leaves could be aligned to the PRSV genome. The reads mapped to the PRSV genome were mostly 21 and 22nt species. Of the aligned reads, 21 and 22nt species constitute 64% (41% and 23% for 21nt and 22nt) and 60% (36% and 24%) in SunUp leaves and PRSV infected AU9 leaves, respectively (Additional file 1: Figure S7). The reads from the SunUp leaves mapped exclusively to the coat protein region of the viral genome suggest active transcription of the transgene that produces sRNA precursors. The reads from the non-transgenic AU9 variety mapped evenly throughout the viral genome (Figure 6).

## Discussions

Our results provide the first report of asymmetric accumulation of purine rich strands in endogenous sRNA populations (Figure 5A, 5B; Additional file 1: Figure S5A, S5B). The endogenous sRNAs are processed from a duplex RNA precursor by an RNase III enzyme dicer. The final dicer product is a short fragment of the RNA duplex formed by Watson-Crick base pairing with a two-nucleotide overhang at the 3’ end. Equal proportions of purine-pyrimidine composition in each pair of sequences were expected according to Chargaff’s rule. Thus, equal proportion of purine rich and pyrimidine rich sequences are expected in the total sRNA population. We found that the nucleotide composition of the cellular sRNA population is highly biased towards purine rich molecules in our papaya sRNA library (Figure 5A, 5B) and in sRNA reads from other plant species obtained from NCBI (Additional file 1: Figure S5A, S5B). Furthermore, the shorter sequences had a high frequency of guanine, whereas adenine was more prevalent in the longer sequences (Figure 5B). We observed the high guanine frequency in 22nt or shorter sequences, while adenine was more frequent in 23nt or longer sequences. This implies that the internal energy of the duplex is maintained by adjusting the ratio of guanine (3 hydrogen bonds) to adenine (two hydrogen bonds) as the sequence length changes.

The highly distorted purine pyrimidine ratio in cellular sRNA population implies two possible scenarios; 1) the cell selectively accumulates purine rich strands and eliminates the pyrimidine rich strands, or 2) more than half of the cellular small RNAs are processed from purine rich single stranded transcripts without having to form a duplex structure. Since there is no known mechanism to process single stranded sRNA, the second scenario is less likely to be the mechanism for such asymmetric distribution.

The accumulation of purine rich sRNAs implies an active strand selection mechanism in the cell. Only one strand of the duplex RNA gets incorporated to RISC and provides sequence specificity to the sRNA targets. The mechanism that selects the guide strand from passenger strand is vaguely known. Since incorporation of the guide strand to RISC is before the target binding, the nucleotide sequence of target cannot be the mechanism to select the guide strand. Asymmetric distribution of internal energy between 5’ and 3’ of the guide strand has been described as a mechanism of strand selection [54,55]. However, different Argonaute proteins appear to have different mechanisms for effector strand selection. AGO1 relies on asymmetric thermodynamic stability between 5’ and 3’ ends, while AGO2 requires mismatches at positions 9 and 10 [56]. Here we present the highly skewed accumulation of purine rich sequences in the cell as a possible alternative mechanism of effector strand selection by the RISC complex.

Technical bias during library preparation, or contamination with some degradation products of other classes of RNA could produce some sort of bias in the deep sequencing libraries. However, both of these scenarios are less likely to have influenced the purine-pyrimidine bias observed here. The contaminant RNAs should be distributed in all size classes rather than accumulating in 20-24nt ranges. In the papaya sRNA libraries, 21 and 24nt reads make ~65% of the 18-33nt total reads, while this goes to ~88% if we use 20-24nt reads for the calculation. This shows that canonical dicer products mainly represent our libraries and the other contaminant RNAs are not enough to influence the observed nucleotide bias. Furthermore, the Arabidopsis sRNA data used for this analysis (GSM253622-25) were AGO pulled reads, which excludes the possibility of contamination from other classes of RNAs to produce the purine-pyrimidine bias. Differential representation of sRNA sequences in a deep sequencing library due to the RNA ligase efficiency has been reported [57,58]. However, these differences are due to the RNA secondary structure, rather than primary sequence. The library preparation protocol we used also acknowledges the differential ligation efficiency towards the end nucleotide of the sRNA sequence [59]. The bias we observed throughout the entire length is less likely to be caused by RNA ligase preference. The purine pyrimidine asymmetry was consistent in the sRNA dataset generated independently by different labs and from different plant species. If, in fact, this asymmetry were due to technical bias, it would imply that we were missing a vast amount of potentially regulatory RNAs in many organisms and the protocol needs to be revisited.

Approximately 25% of the reads in the plant sRNA datasets analyzed had higher pyrimidine content than purine. These reads might represent the un-degraded passenger strand or from unbiased miRNAs (see below) and some contaminant from degradation products of other RNA classes.

However, the purine-pyrimidine bias was not observed in the annotated miRNAs obtained from miRBase [53] and from papaya (Figure 5C). Uracil was the most frequent nucleotide at the 5' end of 21nt miRNA and adenine was most frequent at 5' end of 24nt miRNA. We did not observe any definable pattern of nucleotide conservation along the entire length of miRNAs as was observed in the total cellular sRNA (Additional file 1: Figure S5).

Micro RNAs are evolutionarily ancient than the other classes of regulatory RNAs [15,39,60]. It is also different from other classes of regulatory RNAs on having mismatches and bulges [2,61]. It can be hypothesized that miRNA has acquired independency to purine-pyrimidine ratio over time. We observed that papaya specific miRNAs are more biased towards purine rich strands than the conserved miRNAs, providing more support for evolutionary shift towards purine-pyrimidine equilibrium from siRNAs to miRNA.

The 27% of papaya genome, not represented in the draft genome, contains highly repetitive sequences. Approximately 45% of the unmapped reads from each of the three libraries should be coming from the 27% repetitive regions. The short read sequences often match to multiple loci of the genome. A significant portion of the reads mapped to the available draft sequence should also map to the repetitive sequence of the genome, implying the excessive expression of short RNAs from the repetitive regions of the genome, as observed in other species [9-12]. The 21nt species are preferentially expressed from a small number of highly transcribed genomic loci, while 24nt species are evenly expressed throughout the genome. Five of the nine papaya linkage groups showed at least one location with excessive expression of 21nt species (Figure 4).

Higher accumulation of total reads over unique reads was observed in 21nt species relative to 24nt species (Figure 1A and 1B). This suggests that the 21nt species are transcribed from fewer loci but expressed more. Our data showed higher accumulation of 21 and 22nt species in PRSV infected tissue than uninfected tissues for both unique and total reads. Elevated siRNA accumulation has been observed to the virus-infected plants [37,62,63]. Sequestration of sRNA by virus produced proteins has been observed in plants [64,65]. We observed the accumulation of redundant 21 and 22nt reads in the virus infected leaves, as well as higher expression of unique transcripts indicating that the elevation of these sequences was not the result of sequestration but transcription (Figure 1A). Most of the sRNA reads mapped to the PRSV genome was 21 and 22nt further indicating the virus-induced expression of 21 and 22nt reads (see below).

The SunUp and AU9 provides an opportunity for the comparative study of transgene silencing and virus defense. In plants, sRNA mediated silencing is an important mechanism against virus and transgene invasion [66-68]. The sRNA reads from SunUp leaves were mapped exclusively to the coat protein region of the PRSV genome, suggesting that the coat protein region was enough to induce the host response towards the virus. The AU9 reads however mapped to the entire PRSV genome (Figure 6). The reads mapped to the PRSV genome were predominantly represented by the 21 and 22nt size class (Additional file 1: Figure S7), showing DCL4 (Dicer Like 4) and DCL2 dependent host response against virus infection [61,69,70].

Despite the high abundance of total sRNAs in the cell, only a small portion of the cellular sRNA population is represented by more than one copy (Figure 3). About 85% of the unique sequences in all three papaya libraries were represented by single copy reads. The proportion of the single copy reads in six other plant sRNA datasets generated independently by different labs were also in the same range, implying that this is the nature of cellular sRNA (Additional file 1: Figure S4). Most of the single copy reads map to overlapping genomic loci that are different from others by only a few nucleotides (Additional file 1: Figure S3), showing that the reads are the product of imprecise dicer processing from the common transcript. Transcription of these single copy sequences from overlapping genomic loci suggests that their role in regulation, if any, is at the chromatin level, rather than posttranscriptional regulation at RNA level, which requires more specificity and abundance of the RNA molecule.

We identified 60 miRNA previously not reported in papaya. One conserved miRNA (miR162) was previously reported, in the papaya root transcriptome [45]. It was not found in our library, possibly because of different tissue and developmental phase we have used. We found more papaya specific miRNA than those conserved in other species. The highly conserved and ancient miRNAs show higher expression level than species specific and young miRNA [20,41]. Similar expression bias was observed in papaya miRNA (Table 1).

Of the 60 total annotated miRNA, 24 from 18 families showed homology to the miRNAs from other species, and 36 from 35 families were novel miRNA specific to papaya. This number is much smaller than the numbers reported in other plant species. Although not true for all species, the number of miRNAs appeared to be correlated with the number of whole genome duplication events in those species (Additional file 1: Table S3). We observed fewer miRNAs in papaya than most of other angiosperm species, as previously reported for protein coding genes. This could be partly explained by the lack of whole genome duplication in papaya [44].

A total of 35 of the 60 identified miRNA showed tissue specific expression in papaya. Because of the highly variable nature of miRNA expression in tissues and transient nature of several miRNAs, we couldn’t normalize the qPCR data with any housekeeping genes (miRNA or siRNA), so the qPCR data was normalized to the initial amount of input RNA. The qPCR data are mainly presented for detection purpose and cannot be interpreted as true expression level. We relied on the deep sequencing reads normalized to per million reads to present the miRNA expression level (Table 1). Of the 36 novel miRNAs annotated from papaya, 28 were recorded from only one tissue, indicating the tissue specific function of these new miRNAs.

The complementary strand of miRNAs, miRNA*, degrades after the guide strand in incorporated into the RISC complex. We observed significant accumulation of the miRNA* in PRSV infected leaves (Figure 2), suggesting that it has a potential regulatory function in stressed conditions. Recent studies have pointed that the potential function of miRNA* has been implicated in mammalian cells [71-73]. Increased expression or miRNA* and its regulatory role by targeting mRNA has been shown in plant-micorrhizal symbiosis [74]. In drosophila, AGO2 preferentially selects the miRNA* from the miRNA/miRNA* duplex [56]. AGO2 plays an important role in defense against Flock House Virus (FHV) in drosophila and mosquito [37,75]. We observed elevated accumulation of miRNA* for nine miRNA and seven of them were in PRSV infected leaves, further supporting its role under stressed conditions.

## Conclusions

This is the first report of an asymmetric purine-pyrimidine distribution in the endogenous small RNA population. The sRNAs are generated in the form of an RNA duplex formed by Watson-Crick base pairing. If one of the strands in the duplex is purine rich, the complementary strand should be pyrimidine rich; thus, in the total population, the purine rich strand is expected to be equal to the pyrimidine rich strand, according to Chargaff’s rule. We propose that the observed asymmetric accumulation is due to an active selection mechanism in the cell. Although it needs to be experimentally verified, it is mostly likely to be the mechanism to select the effector strand from the sRNA duplex generated by the dicer enzyme.

The expression of cellular sRNAs varies in different tissues and genomic locations. The majority of cellular sRNAs are represented by only one copy, and they come from overlapping genomic locations. The sRNAs functioning in posttranscriptional gene regulation are expected to have high specificity to the target and are expressed in higher levels. The large number of single copy sRNAs may function in chromatin level gene silencing. Relatively small numbers of sRNA in the cell are expressed in multiple copies and these might function at the post-transcriptional level.

The 21nt and 24nt sRNAs also showed distinct genomic features. The 21nt producing loci are scattered in the genome and expressed excessively from some isolated locations, whereas 24nt species are almost evenly distributed throughout the genome. The 21-22nt sRNAs were highly accumulated in virus-infected tissue, relative to the 23-24nt species. This difference in expression pattern between 21 and 24nt species calls for further investigation on their regulation at the molecular level.

We annotated 60 miRNAs in papaya, of which 24 were conserved in other species, while 36 were not yet reported in other species and may be specific to papaya. Analyzing the annotated miRNA expression in papaya shows higher accumulation of some miRNA* in virus infected tissue. The higher accumulation of the passenger strand compared to the guide strand shows the potential function of these RNA copies in some specific conditions.

## Methods

### Plant materials

The papaya trees were maintained at the Kunia station, Oahu, at the Hawaii Agricultural Research Center. The leaf tissues were collected from one to three week old SunUp plants. The flowers were collected from AU9 female plants one to three weeks after flowering. The PRSV infected leaves were collected from two to three week old AU9 male plants.

### Small RNA extraction, library construction, sequencing

Total RNA was extracted using TRI Reagent (Molecular Research Center) from the three samples. The total RNA was sent to Illumina (Hayward, CA, http://www.illumina.com) for sRNA library construction. According to Illumina, sRNA was gel-sized to a 18-33nt range and the library is constructed using approaches described in [59] with some modifications. Thus constructed library was sequenced with the Sequencing-by-synthesis (SBS) technology on an Illumina Genome Analyzer II. The final sequence was processed to remove 3’ and 5’ adapters and used for downstream analysis. Of the 18–33 nucleotide raw reads, 18-26nt reads were used for the downstream analysis.

### Micro RNA prediction

The miRNA precursor sequence was annotated from deep sequencing reads using the miRDeep algorithm [48] optimized for plant miRNA [48]. The optimum precursor length was set to 250nt. The computationally predicted precursor sequences were further screened on the Rfam web server [76] to remove any false predictions from rRNAs and tRNAs and to confirm the homology of conserved miRNA families. The final screened stem-loop structure (Additional file 1: Figure S8) was detected using the mfold web server [77].

### qRT-PCR analysis of annotated miRNAs

The annotated miRNAs were tested for their expression by stem-loop qRT-PCR [50]. In brief, the stem-loop RT oligos were designed for each miRNA with six nucleotides at the 3’ end complementary to the six nucleotides at the 3’ end of the miRNA (Additional file 1: Table S4). Reverse transcription reactions with the stem-loop oligos form the total RNA extracted from the respective tissues to generate the sRNA cDNA. The cDNAs were used as a template for qRT-PCR. For the qRT-PCR reaction, the DNA oligo complementary to the miRNA sequence, excluding the six nucleotides from the 3’ end, was used as the forward primer while the reverse primer was universal for all miRNAs and complementary to the 5’ end of the stem-loop primer used for cDNA synthesis.

### NCBI small RNA data used for the analysis

*Arabidopsis thaliana*-GSM253622 - GSM253625; *Populus trichocarpa*-GSM717875; *Medicago truncatula*-GSM769274; *Arachis hypogea* (peanut)- GSM769281; *Phaseolus vulgaris*-GSM769290; *Glycine max*- GSM769284.

## Competing interests

The authors declare that they have no competing interests.

## Authors’ contributions

RA, XY, and LL conducted bioinformatics analysis. RA carried out wet lab experiments. RA, LL, RS, and RM wrote the manuscript; RM and QY conceived the study, coordinated all research activities. All authors read and approved the final manuscript.

## Supplementary Material

Additional file 1: Figure S1 Comparison of specific reads in 3 sRNA libraries. **Figure S2.** qPCR verification of predicted miRNAs. **Figure S3.** Schematic representation of mapped sRNA reads showing how the single copy reads differ form each other. **Figure S4.** Percentage of single copy reads in sRNA libraries from 6 plant species. **Figure S5.** Purine-Pyrimidine distribution on sRNA datasets of 6 plant species obtained from NCBI’s GEO database. **Figure S6.** Weblogo picture showing frequency of different nucleotides on miRNAs from miRBase. **Figure S7.** Size distribution of sRNA reads mapped to the PRSV genome. The purple box encloses the total reads from different libraries mapped to the genome. **Figure S8.** Stem loop structure of all annotated miRNAs from papaya. **Table S1.** Deep sequencing reads and mapping to the draft genome. **Table S2.** List of annotated miRNAs in papaya. **Table S3.** Number of miRNAs reported in miRBase from 9 model plant species. **Table S4.** List of stem-loop primers and forward primers used to validate the predicted miRNAs. Click here for file
