# Supplementary material for: Asymmetric purine-pyrimidine distribution in cellular small RNA population of papaya
Source: BMC Genomics. 2012 Dec 5;13:682. doi: 10.1186/1471-2164-13-682 (PMC3582581; doi:10.1186/1471-2164-13-682)
Supplement: Additional file 1: Figure S1 — Comparison of specific reads in 3 sRNA libraries. Figure S2. qPCR verification of predicted miRNAs. Figure S3. Schematic representation of mapped sRNA reads showing how the single copy reads differ form each other. Figure S4. Percentage of single copy reads in sRNA libraries from 6 plant species. Figure S5. Purine-Pyrimidine distribution on sRNA datasets of 6 plant species obtained from NCBI’s GEO database. Figure S6. Weblogo picture showing frequency of different nucleotides on miRNAs from miRBase. Figure S7. Size distribution of sRNA reads mapped to the PRSV genome. The purple box encloses the total reads from different libraries mapped to the genome. Figure S8. Stem loop structure of all annotated miRNAs from papaya. Table S1. Deep sequencing reads and mapping to the draft genome. Table S2. List of annotated miRNAs in papaya. Table S3. Number of miRNAs reported in miRBase from 9 model plant species. Table S4. List of stem-loop primers and forward primers used to validate the predicted miRNAs. [file 1471-2164-13-682-S1.docx]

| **Table S1**. Deep sequencing reads and mapping to the draft genome | | | | | |
| --- | --- | --- | --- | --- | --- |
| **Libraries** | **Total reads** | **Unique reads** | **Reads mapped** | **Percent unique reads** | **Percent unique reads mapped** |
| AU9 Female flower | 4657833 | 2200544 | 1210682 | 47.24% | 55.02% |
| SunUp leaves | 4664779 | 2033600 | 1175392 | 43.59% | 57.80% |
| PRSV infected AU9 male leaves | 4505266 | 1288216 | 700258 | 28.59% | 54.36% |

| **Table S2**- List of annotated miRNAs in papaya | | | | | |
| --- | --- | --- | --- | --- | --- |
| MiR-ID | Chromosome | | Strand | Position | Sequence |
| *Conserved micro RNAs* | | | | | |
| CpmiR156/157a.1 | Supercontig_127 | | + | 309675..309694 | TGACAGAAGAGAGTGAGCAC |
| CpmiR156/157a.2 | Supercontig_46 | | - | 1383706..1383725 |  |
| CpmiR156/157a.3 | Supercontig_7 | | + | 1524402..1524421 |  |
| CpmiR156/157a.4 | Supercontig_28 | | - | 1941022..1941041 |  |
| CpmiR156/157b.1 | Supercontig_170 | | - | 116193..116213 | TTGACAGAAGATAGAGAGCAC |
| CpmiR156/157b.2 | Supercontig_3 | | - | 3349141..3349161 |  |
| CpmiR159a | Supercontig_3 | | - | 3080399..3080419 | TTTGGATTGAAGGGAGCTCTA |
| CpmiR159b | Supercontig_184 | | - | 39569..39588 | CTTGGATTGAAGGGAGCTCC |
| CpmiR159/319 | Supercontig_3 | | - | 3671488..3671507 | ATTGGACTGAAGGGAGCTCC |
| CpmiR160a.1 | Supercontig_102 | | - | 191064..191084 | TGCCTGGCTCCCTGTATGCCA |
| CpmiR160a.2 | Supercontig_192 | | + | 167563..167583 |  |
| CpmiR160a.3 | Supercontig_53 | | + | 453378..453398 |  |
| CpmiR160a.3* | Supercontig_53 | | + | 453438..453458 | GCGTATGAGGAGCCATGCATA |
| CpmiR160b | Supercontig_27 | | + | 1977935..1977955 | TGCCTGGCTCCCTGAATGCCA |
| CpmiR164.1 | Supercontig_327 | | + | 43491..43511 | TGGAGAAGCAGGGCACGTGCA |
| CpmiR164.2 | Supercontig_33 | | + | 1001041..1001061 |  |
| CpmiR164.3 | Supercontig_9 | | + | 1693099..1693119 |  |
| CpmiR165/166a.1 | Supercontig_144 | | - | 273294..273314 | TCGGACCAGGCTTCATTCCCC |
| CpmiR165/166a.2 | Supercontig_52 | | - | 1394502..1394522 |  |
| CpmiR165/166a.3 | Supercontig_58 | | - | 558843..558863 |  |
| CpmiR165/166b | Supercontig_113 | | - | 681120..681140 | TCGGACCAGGCTTCATTCCCG |
| CpmiR166b* | Supercontig_1181 | | + | 12910..12930 | GGAATGTTGGCTGGCTCGAGG |
| CpmiR165/166c | Supercontig_1181 | | + | 12979..12997 | GGACCAGGCTTCATTCCCC |
| CpmiR166c* | Supercontig_1181 | | + | 12910..12930 | GGAATGTTGTTTGGCTCGAGG |
| CpmiR167a.1 | Supercontig_52 | | + | 1081983..1082003 | TGAAGCTGCCAGCATGATCTA |
| CpmiR167a.2 | Supercontig_52 | | + | 1083715..1083735 |  |
| CpmiR167b | Supercontig_44 | | + | 1619889..1619909 | TGAAGCTGCCAGCATGATCTT |
| CpmiR167c | Supercontig_117 | | + | 862509..862530 | TGAAGCTGCCAGCATGATCTGA |
| CpmiR167* | Supercontig_117 | | + | 862566..862586 | ATAGATCATGTGGCAGTTTCA |
| CpmiR169 | Supercontig_119 | | - | 60077..60096 | CAGCCAAGAATGACTTGCCG |
| CpmiR170/171.1 | Supercontig_13 | | - | 901151..901171 | TGATTGAGCCGTGCCAATATC |
| CpmiR170/171.2 | Supercontig_152 | | - | 127577..127597 |  |
| CpmiR170/171.3 | Supercontig_70 | | - | 1070974..1070994 |  |
| CpmiR170/171.4 | Supercontig_87 | | - | 527118..527138 |  |
| CpmiR172.1 | Supercontig_1 | | + | 5257276..5257296 | GGGAATCTTGATGATGCTGCA |
| CpmiR172.2 | Supercontig_81 | | - | 529998..530018 |  |
| CpmiR390.1 | Supercontig_2 | | - | 4098237..4098257 | AAGCTCAGGAGGGATAGCGCC |
| CpmiR390.2 | Supercontig_5 | | + | 1983246..1983266 |  |
| CpmiR390* | Supercontig_5 | | + | 1983298..1983318 | CGCTATCCATCCTGAGTTTCA |
| CpmiR393 | Supercontig_19 | | - | 1807096..1807117 | TCCAAAGGGATCGCATTGATCC |
| CpmiR393* | Supercontig_19 | | - | 1806995..1807015 | ATCATGCTATCCCTTTGGATT |
| CpmiR394.1 | Supercontig_2 | | + | 17563..17582 | TTGGCATTCTGTCCACCTCC |
| CpmiR394.2 | Supercontig_26 | | - | 1711075..1711094 |  |
| CpmiR395.1 | Supercontig_183 | | + | 235122..235141 | TGAAGTGTTTGGGGGAACTC |
| CpmiR395.2 | Supercontig_183 | | + | 234918..234937 |  |
| CpmiR395.3 | Supercontig_183 | | - | 234092..234111 |  |
| CpmiR395.4 | Supercontig_183 | | - | 228239..228258 |  |
| CpmiR395.5 | Supercontig_42 | | + | 185685..185704 |  |
| CpmiR396 | Supercontig_46 | | - | 319993..320013 | TTCCACAGCTTTCTTGAACTG |
| CpmiR396* | Supercontig_46 | | - | 319932..319952 | GTTCAATAAAGCTGTGGGAAG |
| CpmiR408 | Supercontig_812 | | - | 11466..11486 | CTGCACTGCCTCTTCCCTGGC |
| CpmiR408* | Supercontig_812 | | - | 11530..11550 | CTGGGAAGAGGCAGAGCATGG |
| CpmiR535 | Supercontig_8 | | + | 330780..330800 | TGACAACGAGAGAGAGCACGC |
| *Papaya specific micro RNAS* | | | | | |
| CpmiR-novel_01 | | Supercontig_137 | + | 582914..582934 | TGAGAATTATGCGGAGGATGT |
| CpmiR-novel_02 | | Supercontig_34 | + | 1612674..1612694 | GGGACGACATGAGATCACACG |
| CpmiR-novel_03 | | Supercontig_42 | + | 51086..51104 | AGGATTTTGCAGGGTTGAT |
| CpmiR-novel_04 | | Supercontig_282 | - | 4057..4077 | ATTGGAGGACTTTGGGGGAGC |
| CpmiR-novel_05 | | Supercontig_24 | - | 2325224..2325244 | TAAAGTGGAATTGGGATAATA |
| CpmiR-novel_06 | | Supercontig_67 | - | 294023..294043 | TTCGCCAGCCATTCACAAAAT |
| CpmiR-novel_06* | | Supercontig_67 | - | 293979..293999 | TGTGTGAATGGCTTGCGAAAG |
| CpmiR-novel_07 | | Supercontig_80 | - | 269341..269362 | TAAAGATGGTAACAAAGGATAA |
| CpmiR-novel_08.1 | | Supercontig_4264 | - | 5027..5050 | TCCTGGCTGAGGACGGGTGTTGAA |
| CpmiR-novel_08.2 | | Supercontig_8 | + | 2704525..2704548 |  |
| CpmiR-novel_08.3 | | Supercontig_153 | - | 218974..218997 |  |
| CpmiR-novel_08.4 | | Supercontig_2546 | - | 7370..7393 |  |
| CpmiR-novel_08.5 | | Supercontig_54 | - | 1440499..1440522 |  |
| CpmiR-novel_09a | | contig_31148 | + | 885..905 | CGAAAGTAGTGCAATGATGGG |
| CpmiR-novel_09b | | Supercontig_4823 | + | 1038..1058 | CGAAAGTAGTACTAGGATGGG |
| CpmiR-novel_10 | | contig_27314 | - | 10027..10046 | GGTAGTTCGACCGTGAAATT |
| CpmiR-novel_11 | | supercontig_30 | + | 712923..712943 | CTTTTCAAGACTTCAGCTTCA |
| CpmiR-novel_12 | | contig_38619 | - | 1971..1990 | TGGATACTAGTAGGCTGGTT |
| CpmiR-novel_13 | | Supercontig_838 | - | 877..900 | TGAGGTAAGTAGACAGTAAAGGTT |
| CpmiR-novel_14 | | Supercontig_1165 | + | 17175..17198 | GAGAGATGGTGGACAGATCAGGTA |
| CpmiR-novel_15 | | contig_35798 | + | 235..255 | TGGGATTCGGTGCATTAGTGG |
| CpmiR-novel_16 | | contig_39338 | + | 1880..1903 | AGGAATGAACTAGCTAGCAGCGTA |
| CpmiR-novel_17 | | Supercontig_596 | + | 16112..16135 | AACAGTAGAACGAGTTAGAAAGGA |
| CpmiR-novel_18 | | Supercontig_1871 | - | 9345..9368 | ATAAACAGATAGAATGACAGCCTT |
| CpmiR-novel_19 | | Supercontig_6 | - | 2864462..2864485 | AGGAAGACGGTGAGTAGAAGCCAA |
| CpmiR-novel_20 | | Supercontig_242 | - | 16145..16168 | ACTGATACTTGATGAATTTGCATG |
| CpmiR-novel_21 | | Supercontig_124 | + | 5262..5284 | AAGAAGACATGTGGCATGTGCAT |
| CpmiR-novel_22 | | Supercontig_427 | - | 17752..17775 | TGACTGGGTCTGCTGACGTGGCAT |
| CpmiR-novel_23 | | Supercontig_843 | + | 24524..24547 | AGCGAAGGGGACGCCTGAAGACTC |
| CpmiR-novel_24 | | Supercontig_34 | + | 579294..579311 | ATAGTTTGTTTGATGGTA |
| CpmiR-novel_25 | | Supercontig_34 | + | 579293..579310 | TATAGTTTGTTTGATGGT |
| CpmiR-novel_26 | | Supercontig_167 | + | 202896..202919 | AAAACCTGAGTCAGATGATGAGCG |
| CpmiR-novel_27 | | Supercontig_3559 | + | 5241..5260 | TGGATACCAGTAGACAGATA |
| CpmiR-novel_28 | | Supercontig_4 | + | 896938..896958 | TTGGACTGCTAGGTGGCCCAT |
| CpmiR-novel_29.1 | | Supercontig_1287 | - | 14795..14815 | GGGTCGGCGTTGGCATCCTGC |
| CpmiR-novel_29.2 | | Supercontig_5 | + | 792517..792537 |  |
| CpmiR-novel_30 | | Supercontig_4159 | + | 6718..6741 | AGAAGATTGCGAGTAGATACTAGA |
| CpmiR-novel_31 | | Supercontig_6 | - | 1815797..1815820 | TGCACTGTAGAGCCGTATTCGGAC |
| CpmiR-novel_32 | | Supercontig_14 | - | 603957..603977 | GTGCCGTCGCACTGTGACAAG |
| CpmiR-novel_33 | | Supercontig_609 | - | 25620..25641 | CAGAGGAGGAGATGAAGAGGGA |
| CpmiR-novel_34 | | supercontig_13 | + | 267322..267342 | TAACCTGGCTCTGATACCA |
| CpmiR-novel_35 | | Supercontig_15 | + | 526650..526670 | GATCTAAAAAGAGGGGCGTTG |

| **Table S3: Number of miRNAs reported in miRBase from 9 model plant species** | |
| --- | --- |
| Species | Number of miRNAs |
| Papaya | 60 (this study) |
| Grape | 186 |
| Poplar | 237 |
| Medicago | 674 |
| *Arabidopsis thaliana* | 328 |
| *A. lyrata* | 375 |
| Soybean | 395 |
| Rice | 661 |
| Maize | 321 |

| **Table S4.** **List of stem-loop primers and forward primers used to validate the predicted miRNAs**. Uppercase letters are specific to the miRNA while the lowercase letters are stem-loop structures for the stem-loop primer and added to adjust the melting temperature for forward primers. | | | |
| --- | --- | --- | --- |
| **miRNA** | **Sequence** | **Forward primer** | **Stem-loop primer** |
| cpa-MIR156/157 | TGACAGAAGAGAGTGAGCAC | aggcggTGACAGAAGAGAGT | gtcgtatccagtgcagggtccgaggtattcgcactggatacgacGTGCTC |
| cpa-MIR156/157b | TTGACAGAAGATAGAGAGCAC | cccgccgTTGACAGAAGATAG | gtcgtatccagtgcagggtccgaggtattcgcactggatacgacGTGCTCT |
| cpa-MIR159 | TTGACAGAAGATAGAGAGCAC | gcgcggTTGACAGAAGATAGA | gtcgtatccagtgcagggtccgaggtattcgcactggatacgacGTGCTC |
| cpa-MIR159/319 | ATTGGACTGAAGGGAGCTCC | tgccgATTGGACTGAAGGG | gtcgtatccagtgcagggtccgaggtattcgcactggatacgacGGAGCT |
| cpa-MIR160 | TGCCTGGCTCCCTGTATGCCA | cTGCCTGGCTCCCTGT | gtcgtatccagtgcagggtccgaggtattcgcactggatacgacTGGCAT |
| cpa-MIR160* | GCGTGCGAGGAGCCAAGCATG | ctcgtGCGAGGAGCCA | gtcgtatccagtgcagggtccgaggtattcgcactggatacgacCATGCT |
| cpa-MIR164 | TGGAGAAGCAGGGCACGTGCA | ccTGGAGAAGCAGGGCA | gtcgtatccagtgcagggtccgaggtattcgcactggatacgacTGCACG |
| cpa-MIR165/166 | TCGGACCAGGCTTCATTCCCC | ggTCGGACCAGGCTTCA | gtcgtatccagtgcagggtccgaggtattcgcactggatacgacGGGGAA |
| cpa-MIR166* | GGAATGTTGTTTGGCTCGAGG | ccgcGGAATGTTGTTTGGC | gtcgtatccagtgcagggtccgaggtattcgcactggatacgacCCTCGA |
| cpa-MIR167 | TGAAGCTGCCAGCATGATCTA | tcgTGAAGCTGCCAGCAT | gtcgtatccagtgcagggtccgaggtattcgcactggatacgacTAGATC |
| Cpa-MIR167* | ATAGATCATGTGGCAGTTTCA | gcgATAGATCATGTGGCA | gtcgtatccagtgcagggtccgaggtattcgcactggatacgacTGAAAC |
| cpa-MIR169 | CAGCCAAGAATGACTTGCCG | gcggCAGCCAAGAATGAC | gtcgtatccagtgcagggtccgaggtattcgcactggatacgacCGGCAA |
| cpa-MIR170/171 | TGATTGAGCCGTGCCAATATC | gcTGATTGAGCCGTGCC | gtcgtatccagtgcagggtccgaggtattcgcactggatacgacGATATT |
| cpa-MIR172 | GGGAATCTTGATGATGCTGCA | gcgcGGGAATCTTGATGAT | gtcgtatccagtgcagggtccgaggtattcgcactggatacgacTGCAGC |
| cpa-MIR390 | AAGCTCAGGAGGGATAGCGCC | ggcAAGCTCAGGAGGGAT | gtcgtatccagtgcagggtccgaggtattcgcactggatacgacGGCGCT |
| cpa-MIR390* | CGCTATCCATCCTGAGTTTCA | cgcCGCTATCCATCCTGA | gtcgtatccagtgcagggtccgaggtattcgcactggatacgacTGAAAC |
| cpa-MIR393 | TCCAAAGGGATCGCATTGATCC | ccgTCCAAAGGGATCGCAT | gtcgtatccagtgcagggtccgaggtattcgcactggatacgacGGTAGA |
| cpa-MIR393* | ATCATGCTATCCCTTTGGATT | cgcggATCATGCTATCCCTT | gtcgtatccagtgcagggtccgaggtattcgcactggatacgacAATCCA |
| cpa-MIR394 | TTGGCATTCTGTCCACCTCC | aggggTTGGCATTCTGTCC | gtcgtatccagtgcagggtccgaggtattcgcactggatacgacGGAGGT |
| Cpa-MIR395 | TGAAGTGTTTGGGGGAACTC | tcccgTGAAGTGTTTGGGG | gtcgtatccagtgcagggtccgaggtattcgcactggatacgacGAGTTC |
| Cpa-MIR396 | TTCCACAGCTTTCTTGAACTG | tggcgTTCCACAGCTTTCTT | gtcgtatccagtgcagggtccgaggtattcgcactggatacgacCAGTTC |
| Cpa-MIR396* | GTTCAATAAAGCTGTGGGAAG | agcgcgGTTCAATAAAGCTGT | gtcgtatccagtgcagggtccgaggtattcgcactggatacgacCTTCCC |
| Cpa-MIR408 | CTGCACTGCCTCTTCCCTGGC | aggCTGCACTGCCTCTTC | gtcgtatccagtgcagggtccgaggtattcgcactggatacgacGCCAGG |
| Cpa-MIR408* | CTGGGAAGAGGCAGAGCATGG | ggCTGGGAAGAGGCAGA | gtcgtatccagtgcagggtccgaggtattcgcactggatacgacCCATGC |
| Cpa-MIR535 | TGACAACGAGAGAGAGCACGC | gtgcgTGACAACGAGAGAGA | gtcgtatccagtgcagggtccgaggtattcgcactggatacgacGCGTGC |
| Cpa-MIR-novel_01 | TGAGAATTATGCGGAGGATGT | cccgcTGAGAATTATGCGGA | gtcgtatccagtgcagggtccgaggtattcgcactggatacgacACATCC |
| Cpa-MIR-novel_02 | GGGACGACATGAGATCACACG | gccGGGACGACATGAGAT | gtcgtatccagtgcagggtccgaggtattcgcactggatacgacCGTGTC |
| Cpa-MIR-novel_03 | AGGATTTTGCAGGGTTGAT | cgccgAGGATTTTGCAGG | gtcgtatccagtgcagggtccgaggtattcgcactggatacgacATCAAC |
| Cpa-MIR-novel_04 | ATTGGAGGACTTTGGGGGAGC | tggcgATTGGAGGACTTTGG | gtcgtatccagtgcagggtccgaggtattcgcactggatacgacGCTCCC |
| Cpa-MIR-novel_05 | TAAAGTGGAATTGGGATAATA | aggccgTAAAGTGGAATTGGG | gtcgtatccagtgcagggtccgaggtattcgcactggatacgacTATTAT |
| Cpa-MIR-novel_06 | TTCGCCAGCCATTCACAAAAT | accTTCGCCAGCCATTCA | gtcgtatccagtgcagggtccgaggtattcgcactggatacgacATTTTC |
| Cpa-MIR-novel_06* | TGTGTGAATGGCTTGCGAAAG | cgccTGTGTGAATGGCTTG | gtcgtatccagtgcagggtccgaggtattcgcactggatacgacCTTTCG |
| Cpa-MIR-novel_07 | TAAAGATGGTAACAAAGGATAA | tgccgcgTAAAGATGGTAACAAA | gtcgtatccagtgcagggtccgaggtattcgcactggatacgacTTATCC |
| Cpa-MIR-novel_08 | TCCTGGCTGAGGACGGGTGTTGAA | CTGGCTGAGGACGGGT | gtcgtatccagtgcagggtccgaggtattcgcactggatacgacTTCAAC |
| Cpa-MIR-novel_09a | CGAAAGTAGTGCAATGATGGG | tggccCGAAAGTAGTGCAAT | gtcgtatccagtgcagggtccgaggtattcgcactggatacgacCCCATC |
| Cpa-MIR-novel_09b | CGAAAGTAGTACTAGGATGGG | ccgcggCGAAAGTAGTACTAG | gtcgtatccagtgcagggtccgaggtattcgcactggatacgacCCCATC |
| Cpa-MIR-novel_10 | GGTAGTTCGACCGTGAAATT | cgcGGTAGTTCGACCGT | gtcgtatccagtgcagggtccgaggtattcgcactggatacgacAATTTC |
| Cpa-MIR-novel_11 | CTTTTCAAGACTTCAGCTTCA | ccgggcCTTTTCAAGACTTCA | gtcgtatccagtgcagggtccgaggtattcgcactggatacgacTCAAGC |
| Cpa-MIR-novel_12 | TGGATACTAGTAGGCTGGTT | aggcccgTGGATACTAGTAGG | gtcgtatccagtgcagggtccgaggtattcgcactggatacgacAACCAG |
| Cpa-MIR-novel_13 | TGAGGTAAGTAGACAGTAAAGGTT | ggcggTGAGGTAAGTAGACAGTA | gtcgtatccagtgcagggtccgaggtattcgcactggatacgacAAGGTT |
| Cpa-MIR-novel_14 | GAGAGATGGTGGACAGATCAGGTA | ccgGAGAGATGGTGGACAGAT | gtcgtatccagtgcagggtccgaggtattcgcactggatacgacTACCTG |
| Cpa-MIR-novel_15 | TGGGATTCGGTGCATTAGTGG | cccTGGGATTCGGTGCAT | gtcgtatccagtgcagggtccgaggtattcgcactggatacgacCCACTA |
| Cpa-MIR-novel_16 | AGGAATGAACTAGCTAGCAGCGTA | ccgcAGGAATGAACTAGCTAGC | gtcgtatccagtgcagggtccgaggtattcgcactggatacgacTACGCT |
| Cpa-MIR-novel_17 | ATAAACAGATAGAATGACAGCCTT | gcgcgggATAAACAGATAGAATGAC | gtcgtatccagtgcagggtccgaggtattcgcactggatacgacAAGGCT |
| Cpa-MIR-novel_18 | AGGAAGACGGTGAGTAGAAGCCAA | cgcAGGAAGACGGTGAGTAGA | gtcgtatccagtgcagggtccgaggtattcgcactggatacgacTTGGCT |
| Cpa-MIR-novel_19 | ACTGATACTTGATGAATTTGCATG | ccggccACTGATACTTGATGAATT | gtcgtatccagtgcagggtccgaggtattcgcactggatacgacCATGCA |
| Cpa-MIR-novel_20 | AAGAAGACATGTGGCATGTGCAT | tggcAAGAAGACATGTGGCAT | gtcgtatccagtgcagggtccgaggtattcgcactggatacgacATGCAC |
| Cpa-MIR-novel_21 | TGACTGGGTCTGCTGACGTGGCAT | tTGACTGGGTCTGCTGACG | gtcgtatccagtgcagggtccgaggtattcgcactggatacgacATGCCA |
| Cpa-MIR-novel_22 | AGCGAAGGGGACGCCTGAAGACTC | CGAAGGGGACGCCTGA | gtcgtatccagtgcagggtccgaggtattcgcactggatacgacGAGTCT |
| Cpa-MIR-novel_23 | ATAGTTTGTTTGATGGTA | cgcgcgcgATAGTTTGTTTG | gtcgtatccagtgcagggtccgaggtattcgcactggatacgacTACCAT |
| Cpa-MIR-novel_24 | TATAGTTTGTTTGATGGT | tgcgcccgcTATAGTTTGTTT | gtcgtatccagtgcagggtccgaggtattcgcactggatacgacACCATC |
| Cpa-MIR-novel_25 | AAAACCTGAGTCAGATGATGAGCG | gccgAAAACCTGAGTCAGATGA | gtcgtatccagtgcagggtccgaggtattcgcactggatacgacCGCTCA |
| Cpa-MIR-novel_26 | TGGATACCAGTAGACAGATA | tgcccgTGGATACCAGTAGA | gtcgtatccagtgcagggtccgaggtattcgcactggatacgacTATCTG |
| Cpa-MIR-novel_27 | ATATTCAAATGAGAACTTT | gcgcgccgATATTCAAATGAG | gtcgtatccagtgcagggtccgaggtattcgcactggatacgacAAACTT |
| Cpa-MIR-novel_28 | TTGGACTGCTAGGTGGCCCAT | accgTTGGACTGCTAGGTG | gtcgtatccagtgcagggtccgaggtattcgcactggatacgacATGGGC |
| Cpa-MIR-novel_29 | GGGTCGGCGTTGGCATCCTGC | cggcaaGGTCGGCGTTGGCA | gtcgtatccagtgcagggtccgaggtattcgcactggatacgacGCAGGA |
| Cpa-MIR-novel_30 | AGAAGATTGCGAGTAGATACTAGA | agggcAGAAGATTGCGAGTAGAT | gtcgtatccagtgcagggtccgaggtattcgcactggatacgacTCTAGT |
| Cpa-MIR-novel_31 | TGCACTGTAGAGCCGTATTCGGAC | cgtTGCACTGTAGAGCCGTAT | gtcgtatccagtgcagggtccgaggtattcgcactggatacgacGTCCGA |
| Cpa-MIR-novel_32 | GTGCCGTCGCACTGTGACAAG | aGTGCCGTCGCACTGT | gtcgtatccagtgcagggtccgaggtattcgcactggatacgacCTTGTC |
| Cpa-MIR-novel_33 | CAGAGGAGGAGATGAAGAGGGA | acgcCAGAGGAGGAGATGAA | gtcgtatccagtgcagggtccgaggtattcgcactggatacgacTCCCTC |
| Cpa-MIR-novel_34 | TTAGAGGACAGTGGAGTCAAG | accggTTAGAGGACAGTGGA | gtcgtatccagtgcagggtccgaggtattcgcactggatacgacCTTGAC |
| Cpa-MIR-novel_35 | GATCTAAAAAGAGGGGCGTTG | aggcgcGATCTAAAAAGAGGG | gtcgtatccagtgcagggtccgaggtattcgcactggatacgacCAACGC |
|  |  |  |  |
| universal reverse primer | |  | gtgcagggtccgaggt |

**Figure S1**. **Comparison of specific reads 3 sRNA libraries**. Leaf and flower shows more enriched in 24nt small RNA while. * from non normalized reads, † reads normalized to per million reads of respective library.


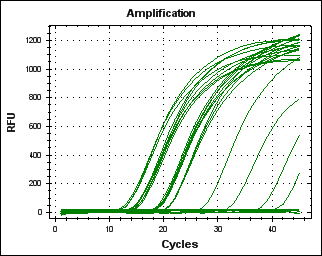

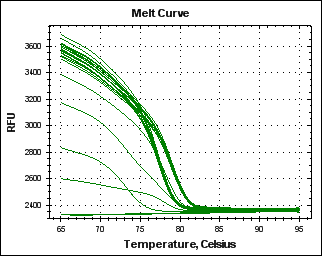

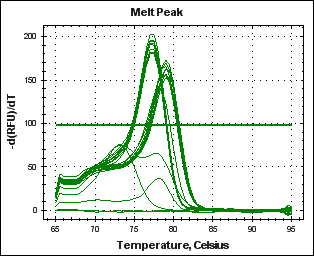


**Figure S2 qPCR verification of predicted miRNAs**. Picture shows the amplification curve of two microRNAs in three different papaya tissues. Each miRNA was tested for three technical replicates. The replicates are grouped in one curve showing that the consistency of the method. The first two ungrouped curves are random primer and with flower cDNA. The last two single curves are no template control. The melting peak data shows two distinct peaks for two miRNA shown.

**Figure S3.** **Schematic representation of mapped sRNA reads showing how the single copy reads differ form each other**. The black line represents the genome, the blue line represents single copy reads, and red line represents multiple copy reads.

.

**Figure S4.** **Percentage of single copy reads in sRNA libraries from 6 plant species.** The data for all 6 species were obtained form NCBI gene expression omnibus. The accession numbers for the data is given in main text.


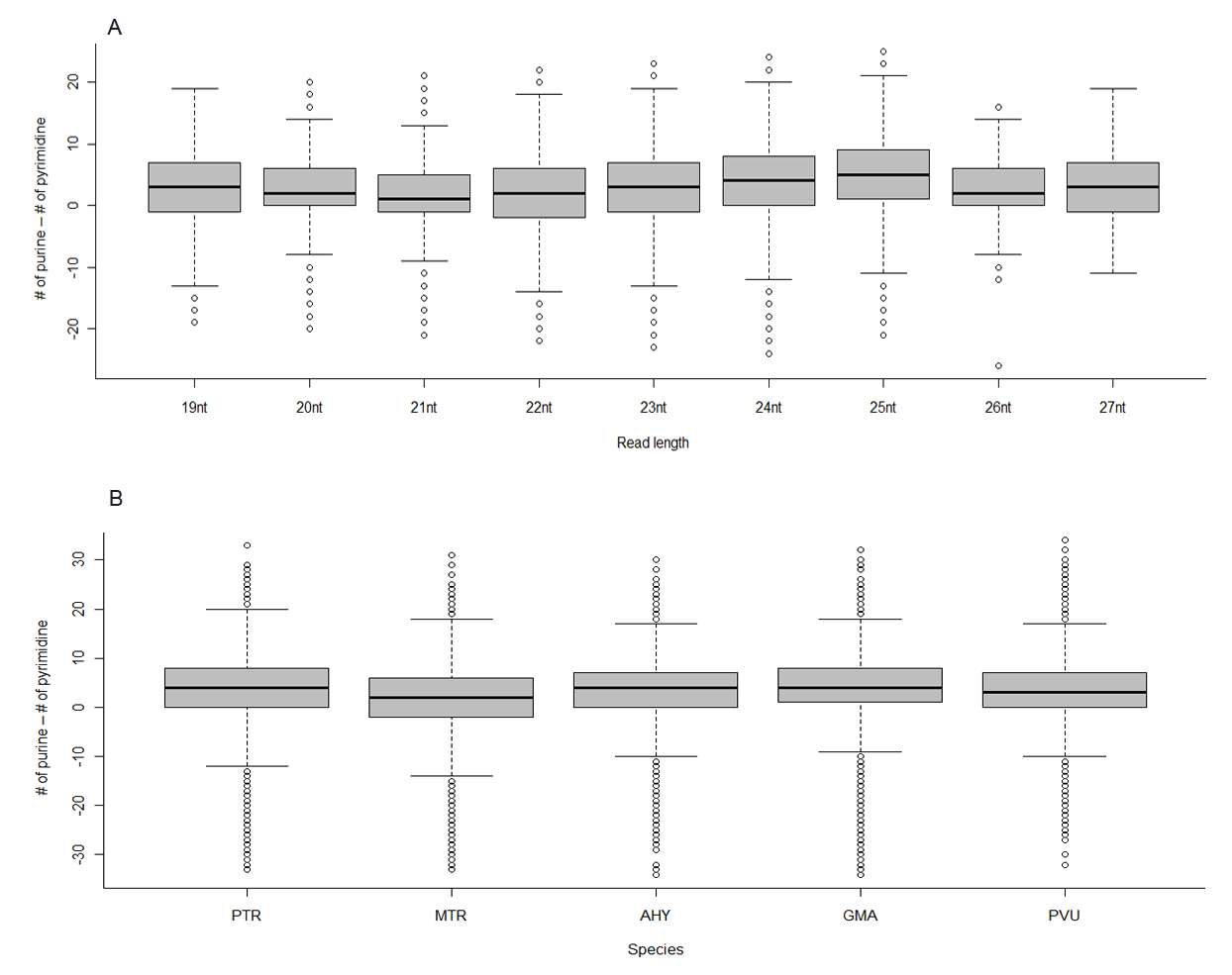


**Figure S5. Purine-Pyrimidine distribution on sRNA datasets of 6-plant species obtained from NCBI’s GEO database**. The accession numbers for the dataset used is provided in main text. The data point above zero line has higher purine content while below zero has higher pyrimidine content.

1. Arabidopsis small RNA data showing the distribution on different size class
2. Purine-Pyrimidine distribution of whole small RNA dataset on 5 plant species. PTR (*Populus* *trichorcarpa*), MTR (*Medicago* *truncatula*), AHY (*Arachis* *hypogea*), GMA (*Glycine* *max*), PVU (*Phaseolous* *vulgaris*).


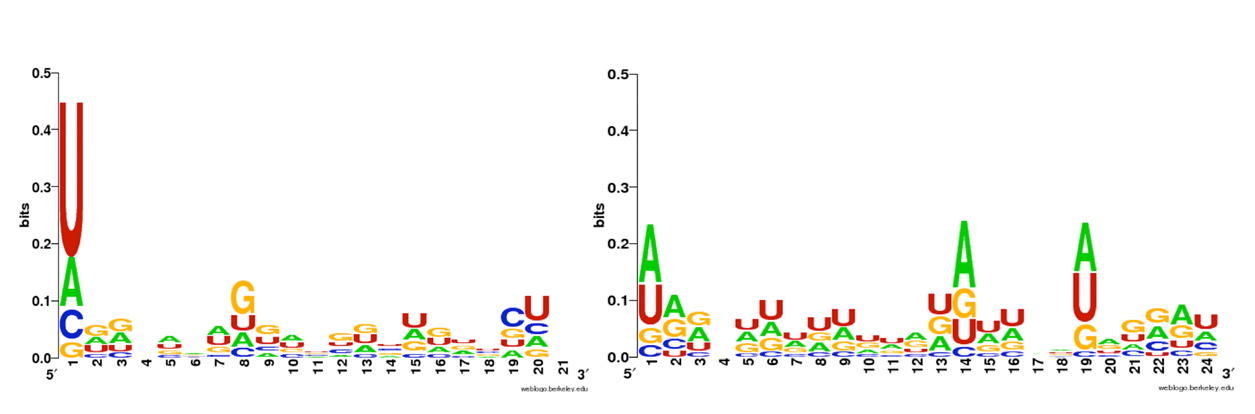


**Figure S6: Weblogo picture showing frequency of different nucleotides on miRNAs from miRBase.** Twenty-one and 24 nucleotide miRNAs from 4 plant species (Arabidopsis, Medicago, Poplar, Rice and) were obtained from miRBase and combined with Papaya miRNAs. The combined 21 and 24nt miRNAs were subjected to the Weblogo web application to generate the figure.

**Figure S7: Size distribution of sRNA reads mapped to the PRSV genome.** The purple box encloses the total reads from different libraries mapped to the genome.


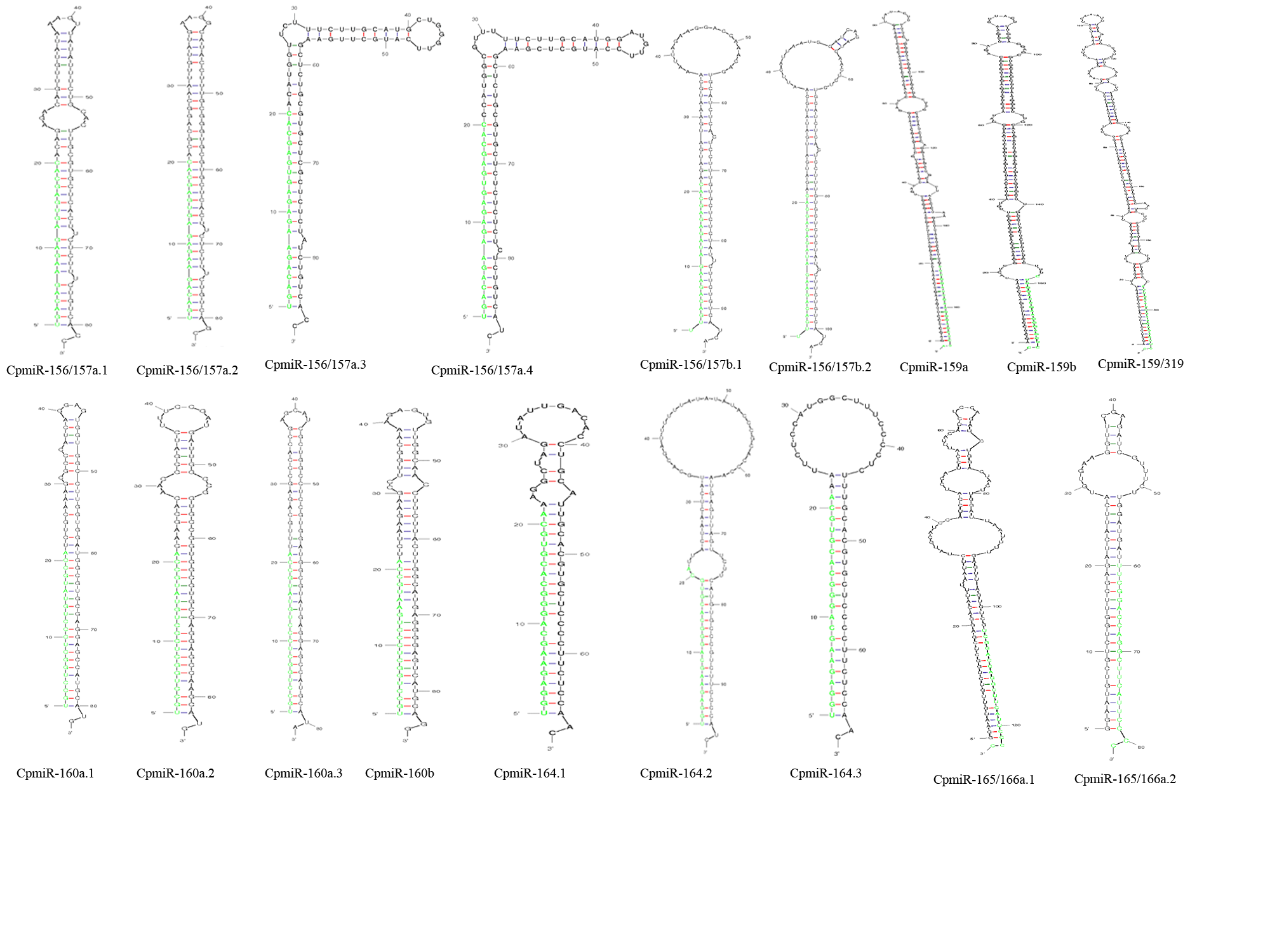

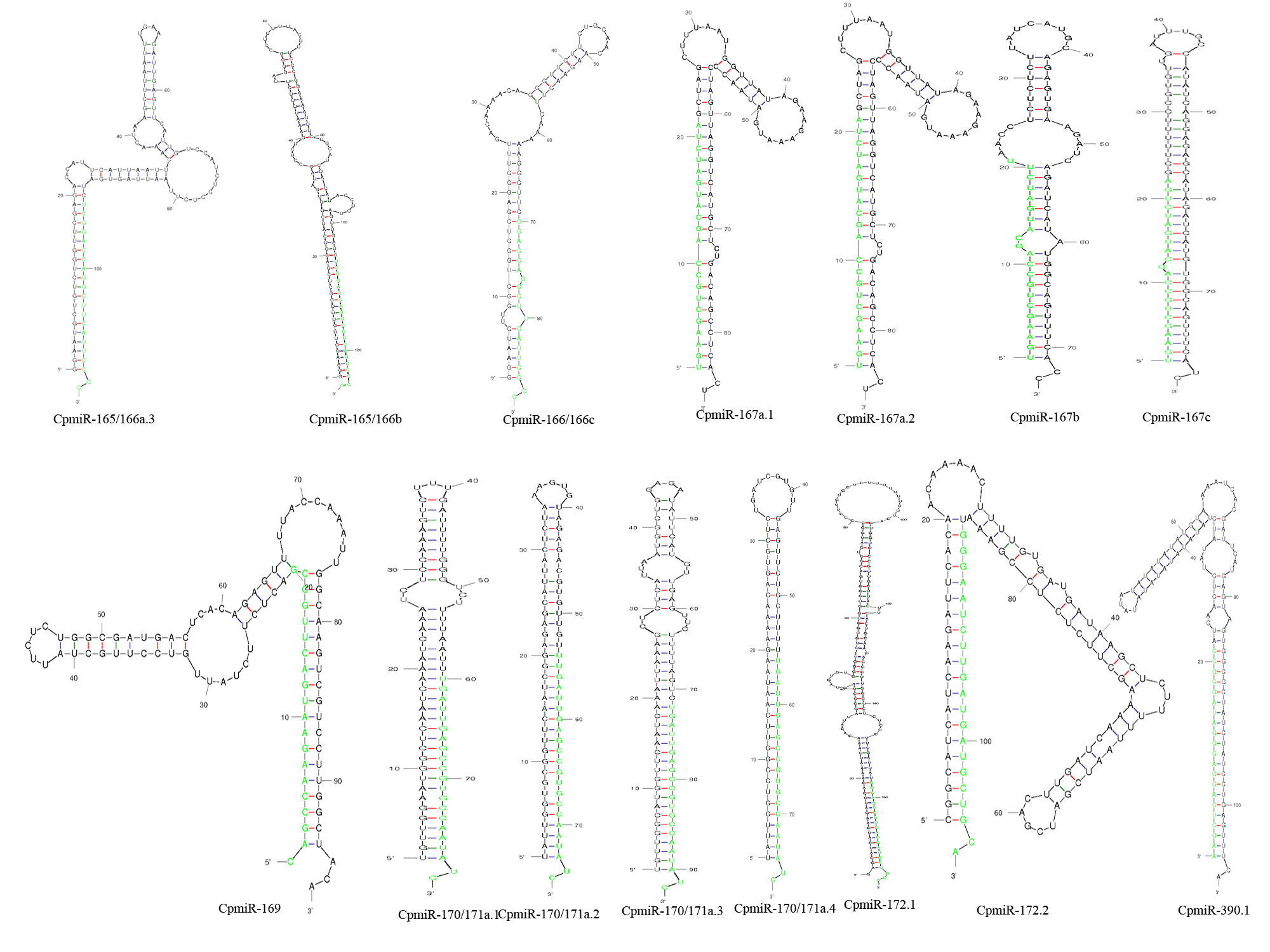


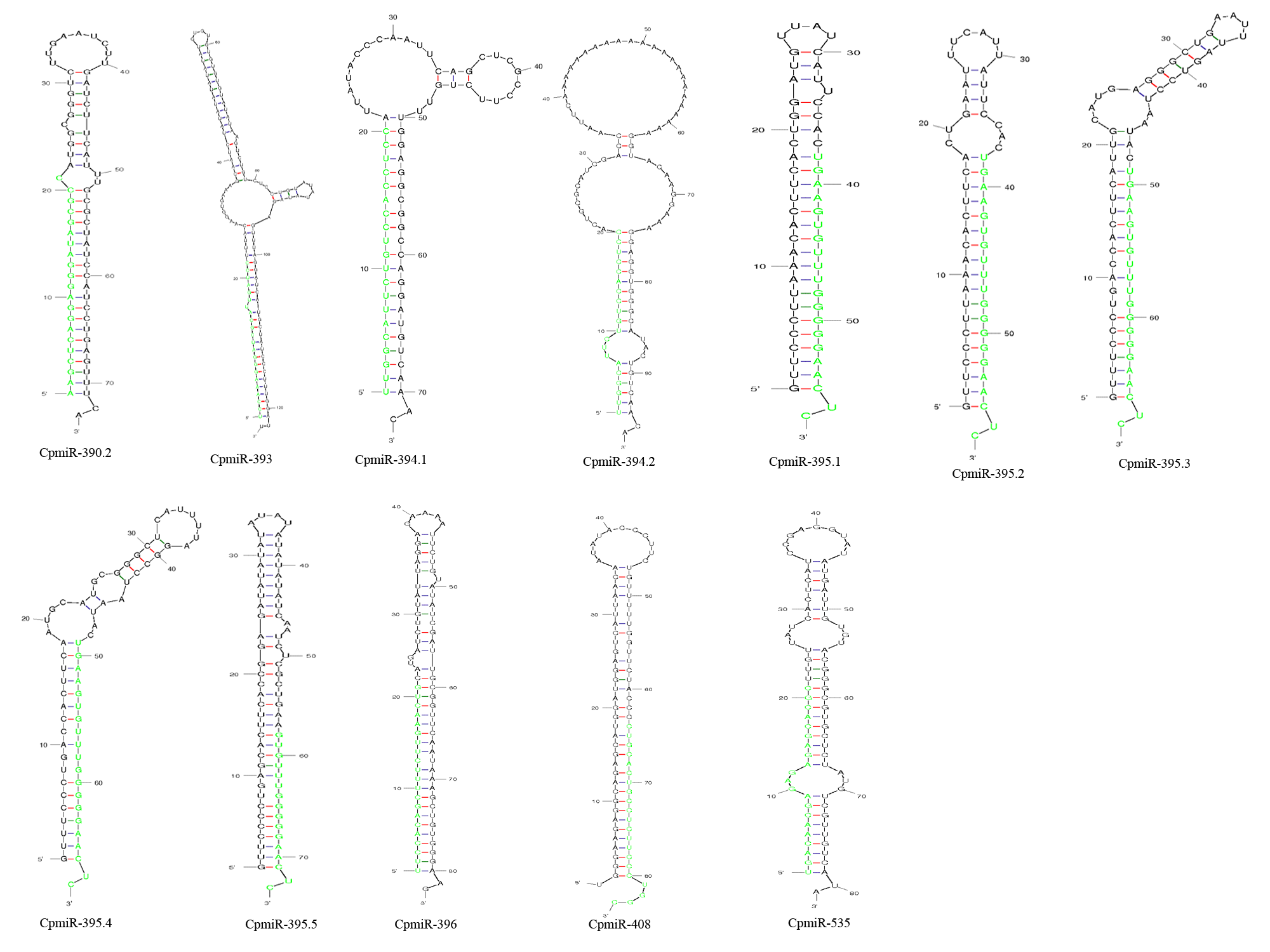


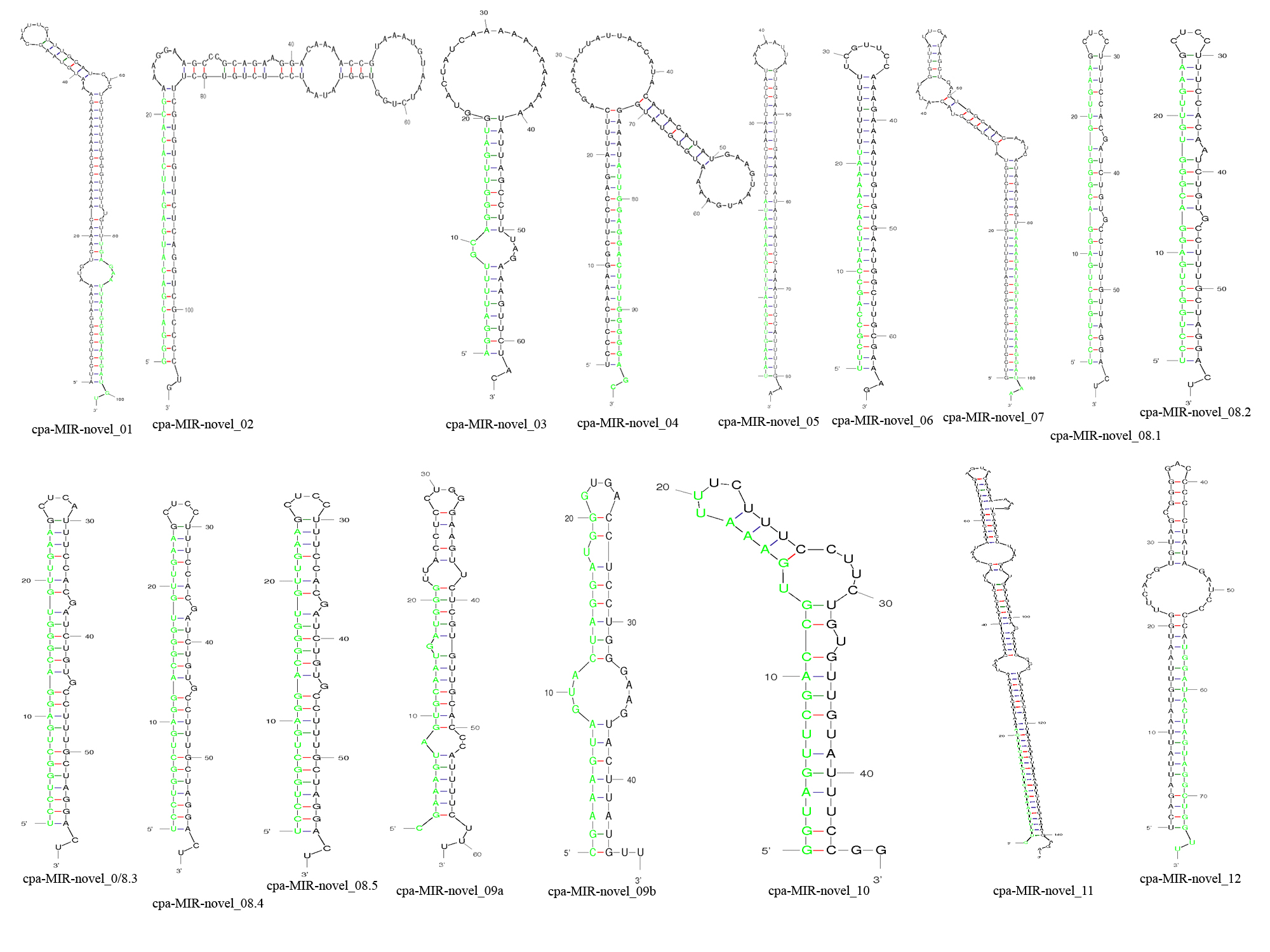


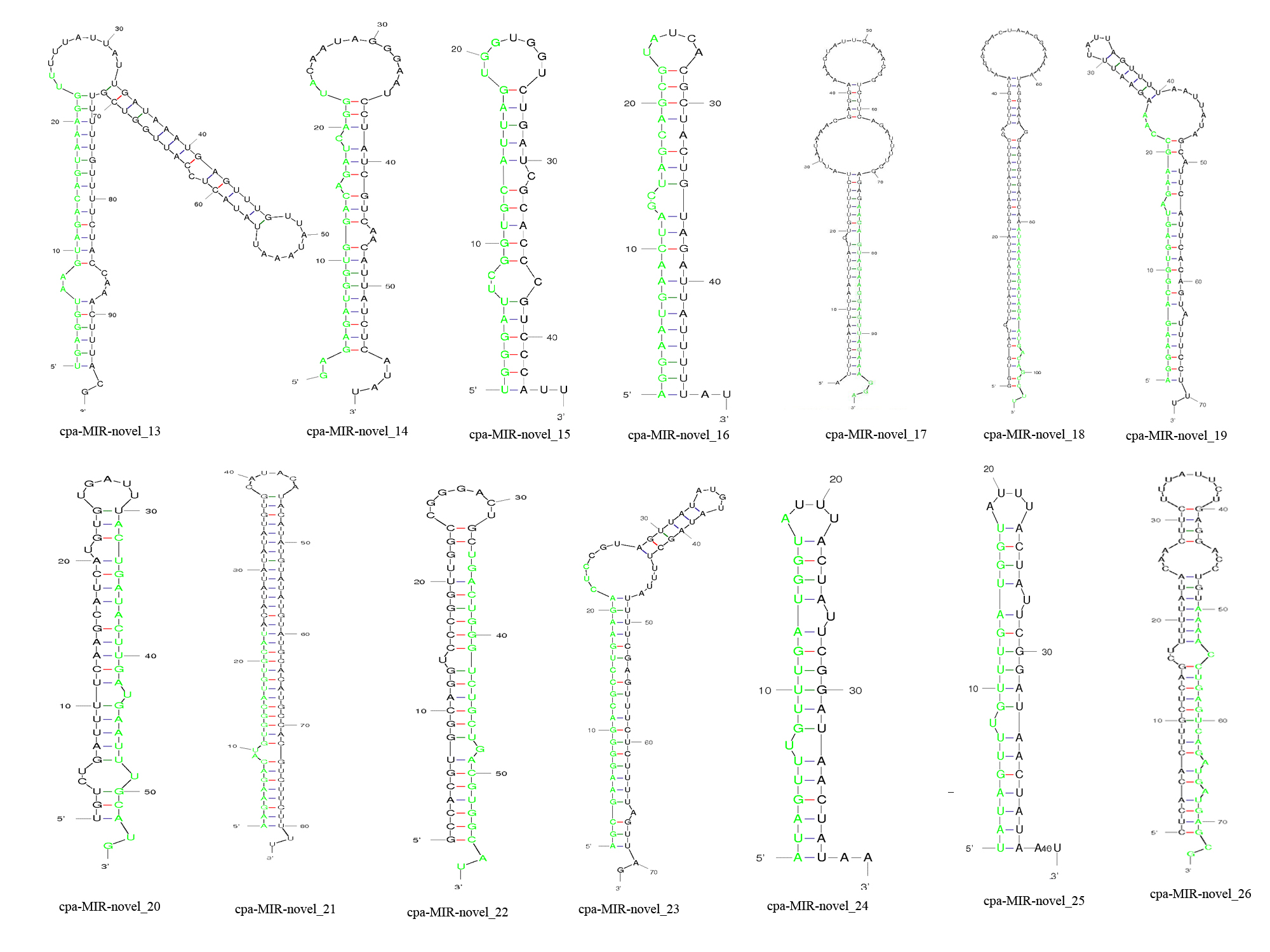


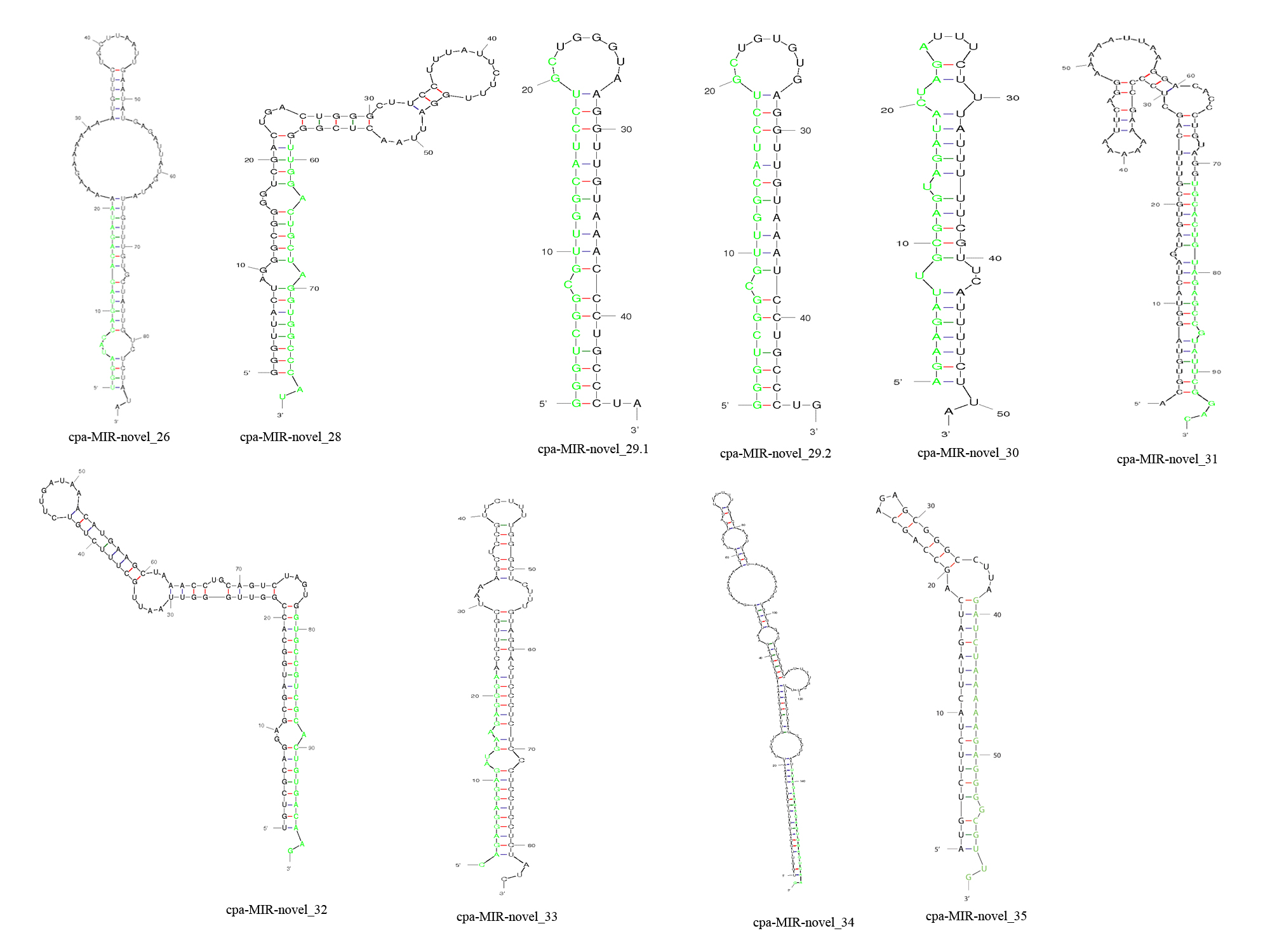


**Figure S8. Stem loop structure of all annotated miRNAs from papaya**. The structure was determined using mfold web server (Zuker 2003) and the secondary structure with lowest free energy was taken.
